# Architecture of the *Escherichia coli* nucleoid

**DOI:** 10.1371/journal.pgen.1008456

**Published:** 2019-12-12

**Authors:** Subhash C. Verma, Zhong Qian, Sankar L. Adhya

**Affiliations:** Laboratory of Molecular Biology, Center for Cancer Research, National Cancer Institute, National Institutes of Health, Bethesda, Maryland, United States of America

## Abstract

How genomes are organized within cells and how the 3D architecture of a genome influences cellular functions are significant questions in biology. A bacterial genomic DNA resides inside cells in a highly condensed and functionally organized form called nucleoid (nucleus-like structure without a nuclear membrane). The *Escherichia coli* chromosome or nucleoid is composed of the genomic DNA, RNA, and protein. The nucleoid forms by condensation and functional arrangement of a single chromosomal DNA with the help of chromosomal architectural proteins and RNA molecules as well as DNA supercoiling. Although a high-resolution structure of a bacterial nucleoid is yet to come, five decades of research has established the following salient features of the *E*. *coli* nucleoid elaborated below: 1) The chromosomal DNA is on the average a negatively supercoiled molecule that is folded as plectonemic loops, which are confined into many independent topological domains due to supercoiling diffusion barriers; 2) The loops spatially organize into megabase size regions called macrodomains, which are defined by more frequent physical interactions among DNA sites within the same macrodomain than between different macrodomains; 3) The condensed and spatially organized DNA takes the form of a helical ellipsoid radially confined in the cell; and 4) The DNA in the chromosome appears to have a condition-dependent 3-D structure that is linked to gene expression so that the nucleoid architecture and gene transcription are tightly interdependent, influencing each other reciprocally. Current advents of high-resolution microscopy, single-molecule analysis and molecular structure determination of the components are expected to reveal the total structure and function of the bacterial nucleoid.

## Introduction

In many bacteria, the chromosome is a single covalently closed (circular) double-stranded DNA molecule that encodes the genetic information in a haploid form. The size of the DNA varies from 500,000 to several million base-pairs (bp) encoding from 500 to several thousand genes depending on the organism. The chromosomal DNA is present in cells in a highly condensed, organized form called nucleoid (nucleus-like), which is not encased by a nuclear membrane as in eukaryotic cells. The isolated nucleoid contains 80% DNA, 10% protein, and 10% RNA by weight [1, 2]. In this exposition, we review our current knowledge about (i) how chromosomal DNA becomes the nucleoid, (ii) the factors involved therein, (iii) what is known about its structure, and (iv) how some of the DNA structural aspects influence gene expression, using the gram-negative bacterium *Escherichia coli* as a model system. We also highlight some related issues that need to be resolved. This exposition is an extension of past reviews on the subject [3, 4].

**Fig 1:**
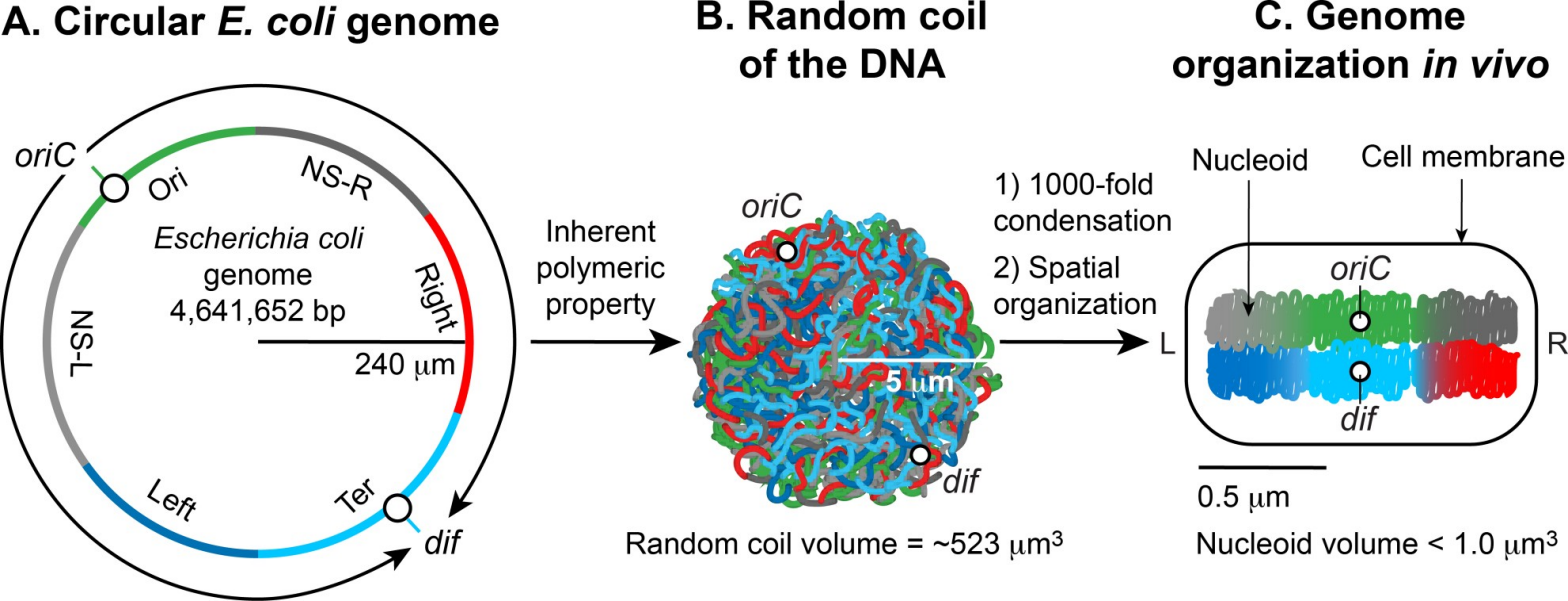
Formation of the *Escherichia coli* nucleoid. **A.** An illustration of an open conformation of the circular genome of *E*. *coli*. Arrows represent bi-directional DNA replication. The genetic position of the origin of bi-directional DNA replication (*oriC*) and the site of chromosome decatenation (*dif*) in the replication termination region (*ter*) are marked. Colors represent specific segments of DNA as discussed in C. **B.** An illustration of a random coil form adopted by the pure circular DNA of *E*. *coli* at thermal equilibrium without supercoils and additional stabilizing factors [5, 6]. **C.** A cartoon of the chromosome of a newly born *E*. *coli* cell. The genomic DNA is not only condensed by 1000-fold compared to its pure random coil form but is also spatially organized. *oriC* and *dif* are localized in the mid-cell, and specific regions of the DNA indicated by colors in A organize into spatially distinct domains. Six spatial domains have been identified in *E*. *coli*. Four domains (Ori, Ter, Left, and Right) are structured and two (NS-right and NS-left) are non-structured (See section 4 of the main text for details). The condensed and organized form of the DNA together with its associated proteins and RNAs is called nucleoid. Drawings are not in scale with each other.

There are two essential aspects of nucleoid formation; condensation of a large DNA into a small cellular space and functional organization of DNA in a three-dimensional form [5, 6]. The haploid circular chromosome in *E*. *coli* consists of ~ 4.6 x 10^6^ bp. If DNA is relaxed in the B form, it would have a circumference of ~1.5 millimeters (0.332 nm x 4.6 x 10^6^) (Fig 1A). However, a large DNA molecule such as the *E*. *coli* chromosomal DNA does not remain a straight rigid molecule in a suspension. Brownian motion will generate curvature and bends in DNA. The maximum length up to which a double-helical DNA remains straight by resisting the bending enforced by Brownian motion is ~50 nm or 150 bp, which is called the persistence length. Thus, pure DNA becomes substantially condensed without any additional factors; at thermal equilibrium, it assumes a random coil form. The random coil of *E*. *coli* chromosomal DNA (Fig 1B) would occupy a volume (4/3 π r^3^) of ~ 523 μm^3^, calculated from the radius of gyration (Rg = (√N a)/√6) where a is the Kuhn length (2 x persistence length), and N is the number of Kuhn length segments in the DNA (total length of the DNA divided by a). Although DNA is already condensed in the random coil form, it still cannot assume the volume of the nucleoid which is less than a micron (Fig 1C). Thus, the inherent property of DNA is not sufficient: additional factors must help condense DNA further on the order of ~10^3^ (volume of the random coil divided by the nucleoid volume). The second essential aspect of nucleoid formation is the functional arrangement of DNA. Chromosomal DNA is not only condensed but also functionally organized in a way that is compatible with DNA transaction processes such as replication, recombination, segregation, and transcription (Fig 1C). Almost five decades of research beginning in 1971 [1], has shown that the final form of the nucleoid arises from a hierarchical organization of DNA. At the smallest scale (1 -kb or less), nucleoid-associated DNA architectural proteins condense and organize DNA by bending, looping, bridging or wrapping DNA. At a larger scale (10 -kb or larger), DNA forms plectonemic loops, a braided form of DNA induced by supercoiling. At the megabase scale, the plectonemic loops coalesce into six spatially organized domains (macrodomains), which are defined by more frequent physical interactions among DNA sites within the same macrodomain than between different macrodomains [7]. Long- and short-range DNA-DNA connections formed within and between the macrodomains contribute to condensation and functional organization. Finally, the nucleoid is a helical ellipsoid with regions of highly condensed DNA at the longitudinal axis [8–10]. We discuss these organizational features of the nucleoid and their molecular basis below.

### DNA condensation and organization by nucleoid-associated proteins (NAPs)

In eukaryotes, genomic DNA is condensed in the form of a repeating array of DNA-protein particles called nucleosomes [11–13].

A nucleosome consists of ~146 bp of DNA wrapped around an octameric complex of the histone proteins. Although bacteria do not have histones, they possess a group of DNA binding proteins referred to as nucleoid-associated proteins (NAPs) that are functionally analogous to histones in a broad sense. NAPs are highly abundant and constitute a significant proportion of the protein component of the nucleoid [14].

A distinctive characteristic of NAPs is their ability to bind DNA in both a specific (either sequence- or structure-specific) and non-sequence specific manner. As a result, NAPs are dual function proteins. The specific binding of NAPs is mostly involved in gene-specific transcription, DNA replication, recombination, and repair. At the peak of their abundance, the number of molecules of many NAPs is several orders of magnitude higher than the number of specific binding sites in the genome. Therefore, it is reasoned that NAPs bind to the chromosomal DNA mostly in the non-sequence specific mode and it is this mode that is crucial for chromosome compaction. It is noteworthy that the so-called non-sequence specific binding of a NAP may not be completely random. There could be low-sequence specificity and or structural specificity due to sequence-dependent DNA conformation or DNA conformation created by other NAPs.

**Fig 2:**
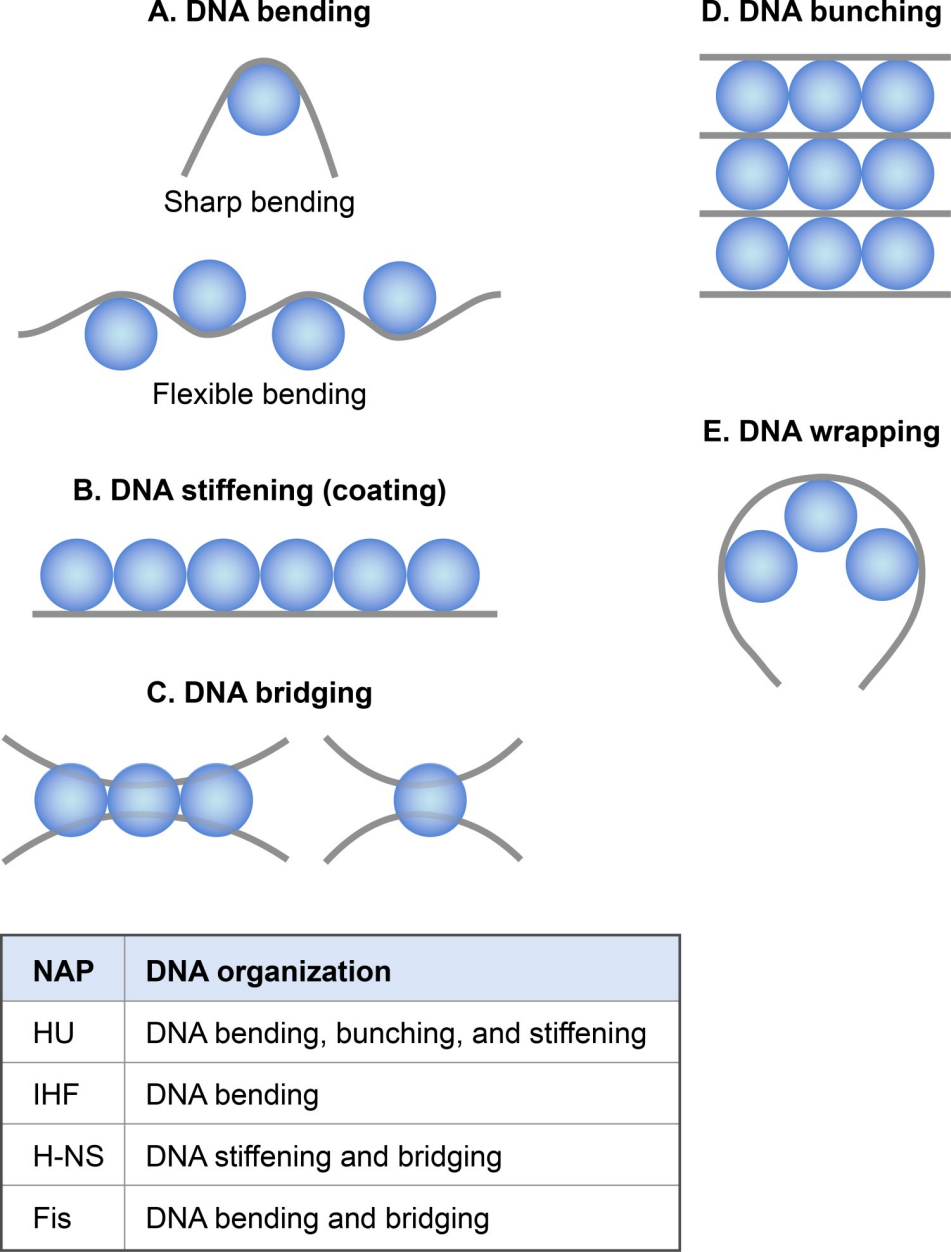
Nucleoid at ≥1 kb scale. **DNA organization by nucleoid-associated proteins (NAPs).** A straight or curved grey line depicts DNA, and blue sphere depicts a NAP. **A.** NAPs organize DNA by bending it. For example, IHF causes sharp DNA bending (bending angle > 160°) upon binding to a specific site, whereas HU introduces flexible bends (bend angles vary between 10–180°). IHF also induce flexible bends at non-sequence-specific sites similar to those induced by HU. Fis bends DNA between 60–75° angle. **B.** In contrast to bending, NAPs can also cause straightening or stiffening of DNA. For example, H-NS spreads along DNA, and as a result, DNA becomes stiff. HU also causes stiffening of DNA at high concentrations (μm range). **C.** Simultaneous binding of a contiguous tract of NAP molecules (left) or a single NAP molecule (right) to a pair of adjacent or distant DNA sites results in DNA bridging. In an example of DNA bridging, a tract of laterally-bound H-NS molecules bridges two adjacent DNA sites. **D**. DNA bunching or bundling refers to DNA organization in which lateral multimerization of HU triggered by the non-sequence-specific binding brings several parallel DNA segments together, like in a bunch of flowers. **E.** NAP molecules bound adjacent to each other can wrap DNA by coherent bending. Fis molecules bound at tandem sites may organize DNA in this manner.

Although molecular mechanisms of how NAPs condense DNA in vivo are not well understood, based on the extensive in vitro studies it appears that NAPs participate in chromosome compaction via the following mechanisms: NAPs induce and stabilize bends in DNA, thus aid in DNA condensation by reducing the persistence length (Fig 2A). NAPs condense DNA by bridging, wrapping, and bunching that could occur between nearby DNA segments or distant DNA segments of the chromosome (Fig 2C, 2D and 2E). Another mechanism by which NAPs participate in chromosome compaction is by constraining negative supercoils in DNA thus contributing to the topological organization of the chromosome (see section 3).

There are at least 12 NAPs identified in *E*. *coli* [15]. Here, we focus on the most extensively studied NAPs, HU, IHF, H-NS, and Fis. Their abundance and DNA binding properties are summarized in Tables 1 and 2. Current models of how each NAP condenses and organizes DNA are discussed in detail below.

**Table 1:** Properties and the abundance of major nucleoid-associated proteins of *E*. *coli*.

Protein	Molecular mass (kDa)	Native functional unit	Abundance^1^ in growth phase	Abundance^1^ in stationary phase
HUα and HUβ	~ 9	Homo- and hetero-dimer	55,000 (23)	30,000 (12.5)
IHFα and IHFβ	~ 11	Heterodimer	12,000 (5)	55,000 (23)
H-NS	~ 15	Homodimer	20,000 (8)	15,000 (6)
Fis	~ 11	Homodimer	60,000 (25)	Undetectable
Dps	~ 19	Dodecamer	6,000 (0.4)	180,000(12.5)

^1^Abundance (molecules/cell) data were taken from [16]. The number in the parenthesis is micromolar concentration calculated using the following formula: (number of native functional units/Avogadro number) x (1/cell volume in liter) x 10^3^. Cell volume in liter (2 x 10^−15^) was determined by assuming volume of the *E*. *coli* cell to be 2 μm^3^.

**Table 2:** DNA binding properties of nucleoid architectural proteins of *E*. *coli*.

Protein	Binding motif	Specific DNA binding affinity^1^	Random DNA binding affinity^1^
HU	A structural motif defined by bends and kinks in DNA [17, 18]	7.5 x 10^−9^ [19]	4.0 x 10^−7^ [19]
H-NS	WATCAANNNNTTR [20]	1.5 x 10^−9^ [21]	1.7 x 10^−6^ [21]
IHF	TCGATAAATT [22]	10–15 x 10^−9^ [23]	6 x 10^−8^ [23]
Fis	GNTYAAAWTTTRANC [24]	0.2–1.0 x 10^−9^ [24, 25]	>8.0 x 10^−6^ [25]
Dps	ND	ND	1.65 x 10^−7^ [26]
MatP	GTGACRNYGTCAC [27]	8.0 x 10^−9^	ND
MukBEF	ND	ND	ND

^1^binding affinity refers to equilibrium dissociation constant (Kd) in molar units (M). ND = not determined

#### HU

**Fig 3:**
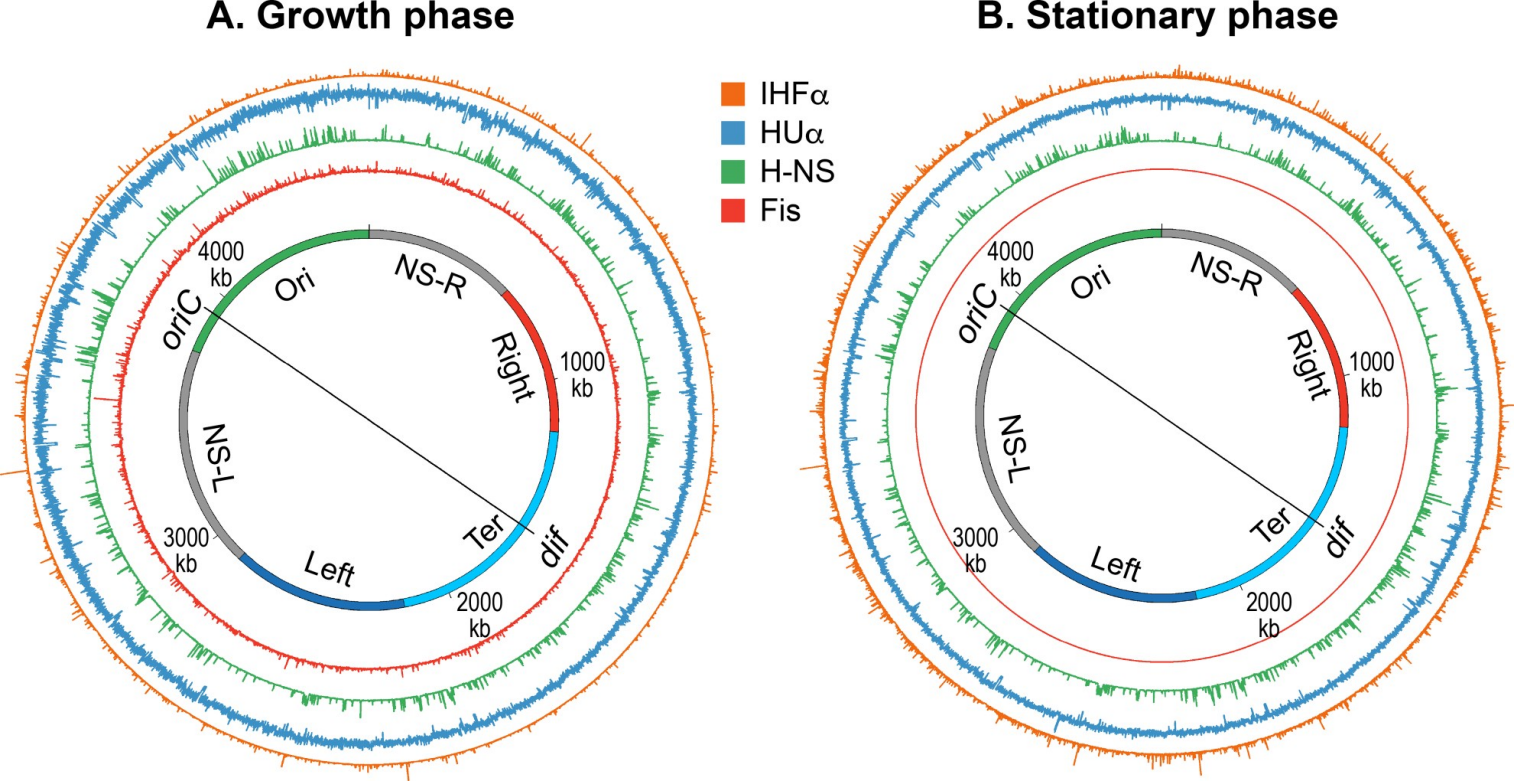
Genome-wide occupancy of nucleoid-associated proteins (NAPs) of *E*. *coli*. **A.** The circular layout of the *E*. *coli* genome (as shown in Fig 1A) additionally depicting the genome occupancy of indicated NAPs in the growth phase. **B.** The genome occupancy of indicated NAPs in the stationary phase. The genome layout is the same as in A. The genome occupancy of each NAP, determined by ChIP-Seq, is plotted as a histogram (bin size 300 bp) in which the bar height is indicative of relative binding enrichment. The figures were prepared in Circos/0.69–6 using the data from [46, 47].

Histone-like protein from *E*. *coli* strain U93 (HU) is an evolutionarily conserved protein in bacteria [28, 29]. HU exists in *E*. *coli* as homo- and heterodimers of two subunits HUα and HUβ sharing 69% amino acid identity [30]. Although it is referred to as a histone-like protein, close functional relatives of HU in eukaryotes are high-mobility group (HMG) proteins, and not histones [31, 32]. HU is a non-sequence specific DNA binding protein. It binds with low-affinity to any linear DNA. However, it preferentially binds with high-affinity to structurally distorted DNA (Table 2) [19, 33–37]. Examples of distorted DNA substrates include cruciform DNA, bulged DNA, dsDNA containing a single-stranded break such as nicks, gaps, or forks. Furthermore, HU specifically binds and stabilizes a protein-mediated DNA loop [38]. In the structurally specific DNA binding mode, HU recognizes a common structural motif defined by bends or kinks created by distortion [17, 18, 39], whereas it binds to a linear DNA by locking the phosphate backbone [40]. While the high-affinity structurally-specific binding is required for specialized functions of HU such as site-specific recombination, DNA repair, DNA replication initiation, and gene regulation [41–43], it appears that the low-affinity general binding is involved in DNA condensation [40]. In chromatin-immunoprecipitation coupled with DNA sequencing ( ChIP-Seq), HU does not reveal any specific binding events. Instead, it displays a uniform binding across the genome presumably reflecting its mostly weak, non-sequence specific binding, thus masking the high-affinity binding in vivo (Fig 3).

In strains lacking HU, the nucleoid is "decondensed", consistent with a role of HU in DNA compaction [44]. The following in vitro studies suggest possible mechanisms of how HU might condense and organize DNA in vivo. Not only HU binds stably to distorted DNA with bends, but it also induces flexible bends even in a linear DNA at less than 100 nM concentration (Fig 2A) [45]. In contrast, HU shows the opposite architectural effect on DNA at higher physiologically-relevant concentrations [40, 45]. It forms rigid nucleoprotein filaments causing the straitening of DNA and not the bending (Fig 2B). The filaments can further form a DNA network (DNA bunching) expandable both laterally and medially because of the HU-HU multimerization triggered by the non-sequence-specific DNA binding (Fig 2D) [40].

How are these behaviors of HU relevant inside the cell? The formation of filaments requires high-density binding of HU on DNA, one HU dimer per 9–20 bp DNA [40, 45]. But there is only one HU dimer every ~150 bp of the chromosomal DNA based on the estimated abundance of 30,000 HU dimers per cell (4600000 bp /30,000) [16]. This indicates that flexible bends are more likely to occur in vivo. The flexible bending would cause condensation due to a reduction in the persistence length of DNA as shown by magnetic tweezers experiments [45], which allow studying condensation of a single DNA molecule by a DNA binding protein [48]. However, because of the cooperativity, the rigid filaments and networks could form in some regions in the chromosome. The filament formation alone does not induce condensation [45], but DNA networking or bunching can substantially contribute to condensation by bringing distant or nearby chromosome segments together [40].

#### IHF

Integration host factor (IHF) is structurally almost identical to HU [49] but behaves differently from HU in many aspects. Unlike HU, which preferentially binds to a structural motif regardless of the sequence, IHF preferentially binds to a specific DNA sequence even though the specificity arises through the sequence-dependent DNA structure and deformability. The specific binding of IHF at cognate sites bends DNA sharply by >160° bend angle [49]. An occurrence of the cognate sequence motif is about 3000 in the *E*. *coli* genome [47]. The estimated abundance of IHF in the growth phase is about 6000 dimers per cell (Table 1). Assuming that one IHF dimer binds to a single motif and nucleoid contains more than one genome equivalent during the exponential growth phase, most of the IHF molecules would occupy specific sites in the genome and likely only condense DNA by inducing sharp bending (Fig 2A).

Besides preferential binding to a specific DNA sequence, IHF also binds to DNA in a non-sequence specific manner with the affinities similar to HU (Table 2). The role of the non-specific binding of IHF in DNA condensation appears to be critical in the stationary phase because the IHF abundance increases by five-fold in the stationary phase (Table 1) and the additional IHF dimers would likely bind the chromosomal DNA non-specifically [16, 50, 51]. Unlike HU, IHF does not form thick rigid filaments at higher concentrations. Instead, its non-specific binding also induces DNA bending albeit the degree of bending is much smaller than that at specific sites and is similar to the flexible bending induced by HU in a linear DNA at low concentrations [52]. In vitro, the bending induced by non-specific binding of IHF can cause DNA condensation and promotes the formation of higher-order nucleoprotein complexes depending on the concentrations of potassium chloride and magnesium chloride [52]. Whether the higher-order DNA organization by IHF occurs in vivo needs further investigation.

#### H-NS

A distinguishable feature of histone-like or heat-stable nucleoid structuring protein (H-NS) [53–56] from other NAPs is the ability to switch from the homodimeric form at relatively low concentrations (<1 x 10^−5^ M) to an oligomeric state at higher levels [57, 58]. Because of oligomerization properties, H-NS spreads laterally along AT-rich DNA in a nucleation reaction, where high-affinity sites function as nucleation centers [21, 59, 60]. The spreading of H-NS on DNA results in two opposite outcomes depending on the magnesium concentration in the reaction (Fig 2C). At low magnesium concentration (< 2 mM), H-NS forms rigid nucleoprotein filaments whereas it forms inter- and intra-molecular bridges at higher magnesium concentrations (> 5 mM) [61–65]. The formation of rigid filaments results in the straightening of DNA (Fig 2B) with no condensation whereas the bridging causes substantial DNA folding [64]. Analysis of H-NS binding in the genome by ChIP-Seq assays provided indirect evidence for the spreading of H-NS on DNA in vivo. H-NS binds selectively to 458 regions in the genome [46]. Although H-NS has been demonstrated to prefer curved DNA formed by repeated A-tracks in DNA sequences [59, 66] the basis of the selective binding is the presence of a conserved sequence motif found in AT-rich regions (Table 2) [20]. More importantly, the frequent occurrence of the sequence motif within an H-NS binding region that can re-enforce the cooperative protein-protein interactions, and the unusually long length of the binding region are consistent with the spreading of the protein. Which of the two outcomes, the filament formation or DNA bridging, is prevalent in vivo? If the physiological concentration of magnesium inside cells is uniformly low (< 5 mM) [67], H-NS would form rigid nucleoprotein filaments in vivo. Alternatively, if there is an uneven distribution of magnesium in the cell, it could promote both DNA bridging and stiffening but in different regions of the nucleoid.

Furthermore, H-NS is best known as a global gene silencer that preferentially inhibits transcription of horizontally transferred genes and it is the rigid filament that leads to gene silencing [68, 69]. Taken together, it appears that the formation of rigid filaments is the most likely outcome of H-NS-DNA interactions in vivo that leads to gene silencing but does not induce DNA condensation. Consistently, the absence of H-NS does not change the nucleoid volume [70]. However, *E*. *coli* may experience high-magnesium concentration under some environmental conditions. In such conditions, H-NS can switch from its filament inducing form to the bridge inducing form that contributes to DNA condensation and organization.

#### Fis

Factor for Inversion Stimulation (Fis) is a sequence-specific DNA binding protein that binds to specific DNA sequences containing a 15-bp symmetric motif (Table 2) [24, 25, 71]. Like IHF, Fis induces DNA bending at cognate sites. The ability to bend DNA is apparent in the structure of Fis homodimer. A Fis homodimer possesses two helix-turn-helix (HTH) motifs, one from each monomer. An HTH motif typically recognizes the DNA major groove. However, the distance between the DNA recognition helices of the two HTH motifs in the Fis homodimer is 25 A°, which is ~ 8 A° shorter than the pitch of a canonical B-DNA, indicating that the protein must bend or twist DNA to bind stably [72, 73]. Consistently, the crystal structure of Fis-DNA complexes shows that the distance between the recognition helices remains unchanged whereas DNA curves in the range of 60–75° bend angles [25]. There are 1464 Fis binding regions distributed across the *E*. *coli* genome and a binding motif, identified computationally, matches with the known 15-bp motif [46, 74]. Specific binding of Fis at such sites would induce bends in DNA, thus contribute to DNA condensation by reducing persistence length of DNA. Furthermore, many Fis binding sites occur in tandem such as those in the stable RNA promoters, e.g., *P1* promoter of rRNA operon *rrnB*. The coherent bending by Fis at the tandem sites is likely to create a DNA micro-loop (Fig 2E) that can further contribute to DNA condensation [75].

Besides high-affinity specific binding to cognate sites, Fis can bind to a random DNA sequence (Table 2). The non-specific DNA binding is significant because Fis is as abundant as HU in the growth phase (Table 1). Therefore, most of Fis molecules are expected to bind DNA in a non-sequence specific manner. Magnetic tweezers experiments show that this non-specific binding of Fis can contribute to DNA condensation and organization [76, 77]. Fis causes mild condensation of a single DNA molecule at <1 mM but induces substantial folding through the formation of DNA loops of an average size of ~800-bp at >1 mM. The loops in magnetic tweezers experiments are distinct from the micro-loops created by coherent DNA bending at cognate sites, as they require the formation of high-density DNA-protein complexes achieved by sequence-independent binding. Although occurrence of such loops in vivo remains to be demonstrated, high-density binding of Fis may occur in vivo through the concerted action of both specific and non-specific binding. The in-tandem occurrence of specific sites might initiate a nucleation reaction similar to that of H-NS, and then non-specific binding would lead to the formation of localized high-density Fis arrays. The bridging between these localized regions (Fig 2C) can create large DNA loops [77]. Fis is exclusively present in the growth phase and not in the stationary phase [78, 79]. Thus, any role in chromosomal condensation by Fis must be specific to growing cells.

### DNA condensation and organization by nucleoid-associated RNAs (naRNAs)

Early studies examining the effect of RNase A treatment on isolated nucleoids indicated that RNA participated in the stabilization of the nucleoid in the condensed state [80]. Moreover, treatment with RNase A disrupted the DNA fibers into thinner fibers, as observed by atomic force microscopy of the nucleoid using the “on-substrate lysis procedure” [81]. These findings demonstrated the participation of RNA in the nucleoid structure, but the identity of the RNA molecule(s) remained unknown until recently [44]. Most of the studies on HU focused on its DNA binding. However, HU also binds to dsRNA and RNA-DNA hybrids with a lower affinity similar to that with a linear dsDNA [82]. Moreover, HU preferentially binds to RNA containing secondary structures and an RNA-DNA hybrid in which the RNA contains a nick or overhang [82, 83]. The binding affinities of HU with these RNA substrates are similar to those with which it binds to distorted DNA. An immunoprecipitation of HU-bound RNA coupled to reverse transcription and microarray (RIP-Chip) study as well as an analysis of RNA from purified intact nucleoids identified nucleoid-associated RNA molecules that interact with HU [44]. Several of them are non-coding RNAs, and one such RNA named naRNA4 (nucleoid-associated RNA 4), is encoded in a repetitive extragenic palindrome (REP325). In a strain lacking REP325, the nucleoid is decondensed as it is in a strain lacking HU [44]. naRNA4 most likely participate in DNA condensation by connecting DNA segments in the presence of HU [84]. Recent studies provide insights into the molecular mechanism of how naRNA4 establishes DNA-DNA connections. The RNA targets regions of DNA containing cruciform structures and forms an RNA-DNA complex that is critical for establishing DNA-DNA connections [85]. Surprisingly, although HU helps in the formation of the complex, it is not present in the final complex, indicating its potential role as a catalyst (chaperone). The nature of the RNA-DNA complex remains puzzling because the formation of the complex does not involve extensive Watson/Crick base pairing but is sensitive to RNase H, which cleaves RNA in an RNA-DNA hybrid. Moreover, the complex binds to an antibody specific to RNA-DNA hybrids.

### DNA condensation and organization by supercoiling

#### Supercoiling

**Fig 4:**
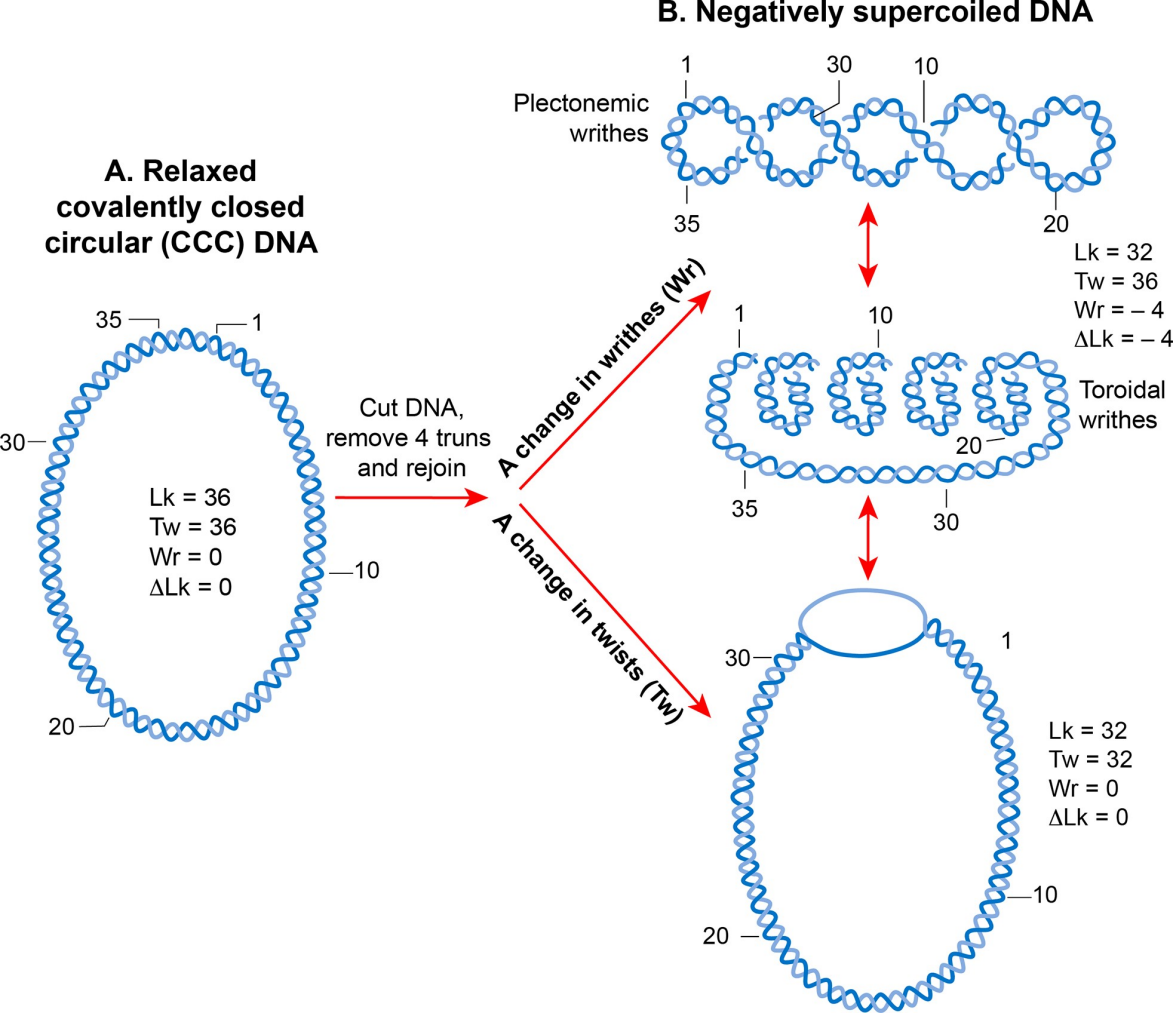
DNA supercoiling. **A.** A linear double-stranded DNA becomes a topologically constrained molecule if the two ends are covalently joined, forming a circle. Rules of DNA topology are explained using such a molecule (ccc-DNA) in which a numerical parameter called the linking number (Lk) defines the topology. Lk is a mathematical sum of two geometric parameters, twist (Tw) and writhe (Wr). A twist is the crossing of two strands, and writhe is coiling of the DNA double helix on its axis that requires bending. Lk is always an integer and remains invariant no matter how much the two strands are deformed. It can only be changed by introducing a break in one or both DNA strands by DNA metabolic enzymes called topoisomerases. **B.** A torsional strain created by a change in Lk of a relaxed, topologically constrained DNA manifests in the form of DNA supercoiling. A decrease in Lk (Lk<Lk_0_) induces negative supercoiling whereas an increase in Lk (Lk>Lk_0_) induces positive supercoiling. Only negative supercoiling is depicted here. For example, if a cut is introduced into a ccc-DNA and four turns are removed before rejoining the two strands, the DNA becomes negatively supercoiled with a decrease in the number of twists or writhe or both. Writhe can adopt two types of geometric structures called plectoneme and toroid. Plectonemes are characterized by the interwinding of the DNA double helix and an apical loop, whereas spiraling of DNA double helix around an axis forms toroids.

Because of its helical structure, a double-stranded DNA molecule becomes topologically constrained in the covalently closed circular form which eliminates the rotation of the free ends [86]. The number of times the two strands cross each other in a topologically constrained DNA is called the linking number (Lk), which is equivalent to the number of helical turns or twists in a circular molecule (Fig 4). The Lk of a topological DNA remains invariant, no matter how the DNA molecule is deformed, as long as neither strand is broken.

The Lk of DNA in the relaxed form is defined as Lk_0_. For any DNA, Lk_0_ can be calculated by dividing the length (in bp) of the DNA by the number of bp per helical turn. This is equal to 10.4 bp for the relaxed B-form DNA. Any deviation from Lk_0_ causes supercoiling in DNA. A decrease in the linking number (Lk<Lk_0_) creates negative supercoiling (Fig 4) whereas an increase in the linking number (Lk>Lk_0_) creates positive supercoiling (see [87, 88] for more detail of supercoiling).

The supercoiled state (when Lk is not equal to Lk_0_) results in a transition in DNA structure that can manifest as a change in the number of twists (negative <10.4 bp/turn, positive >10.4 bp per turn) and/or in the formation of writhes, called supercoils (Fig 4). Thus, Lk is mathematically defined as a sign dependent sum of the two geometric parameters, twist and writhe. A quantitative measure of supercoiling that is independent of the size of DNA molecules is the supercoiling density (σ) where σ = ΔLk/Lk_0_.

**Fig 5:**
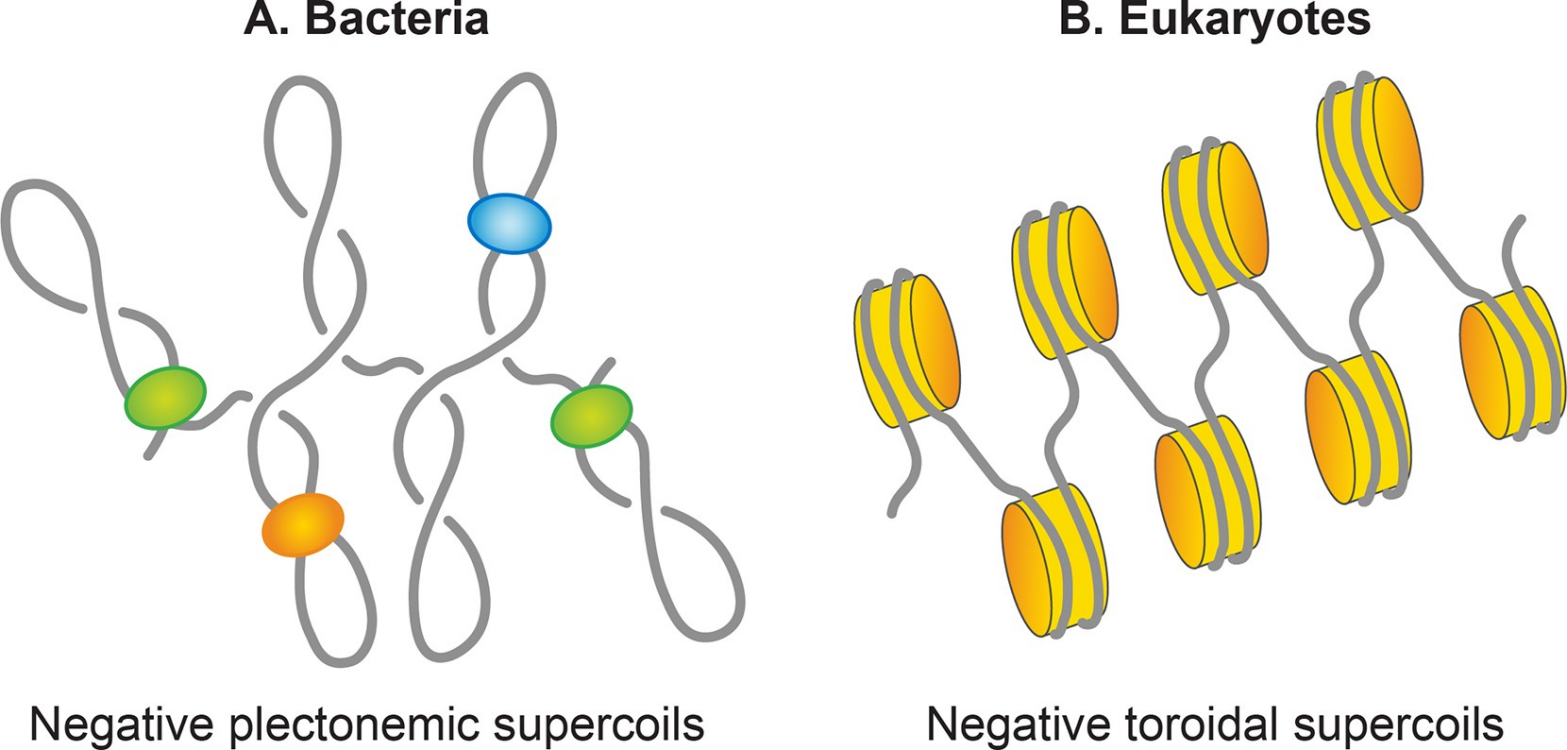
Basic units of genomic organization in bacteria and eukaryotes. **A.** A bacterial genome organizes as plectonemic supercoils. Half of the supercoils are present in free form, and nucleoid-associated proteins (NAPs), shown as colored spheres, restrain the remaining half. **B.** In contrast, a eukaryotic genome organizes as toroidal supercoils, induced by the wrapping of DNA around histone proteins (orange color). An octamer of histones with 146 wrapped DNA refers to as nucleosome, and the genome organizes into a repeating array of nucleosomes.

Writhes can adopt two structures; plectoneme and solenoid or toroid (Fig 4). A plectonemic structure arises from the interwinding of the helical axis (Fig 4). Toroidal supercoils originate when DNA forms several spirals, around an axis and not intersecting with each other, like those in a telephone cord. The writhes in the plectonemes form are right- and left-handed in negatively or positively supercoiled DNA, respectively. The handedness of the toroidal supercoils is opposite to those of plectonemes. Both plectonemes and toroidal supercoils can be either in a free form or restrained in a bound form with proteins. The best example of the bound toroidal supercoiling in biology is the eukaryotic nucleosome in which DNA wraps around histones (Fig 5) [12].

#### The *E*. *coli* genome is organized as plectonemic supercoils

In most bacteria, DNA is present in a supercoiled form. The circular nature of the *E*. *coli* chromosome makes it a topologically constrained molecule that is mostly negatively supercoiled with an estimated average supercoiling density (σ) of -0.05 [89]. In the eukaryotic chromatin, DNA is found mainly in the toroidal form that is restrained and defined by histones through the formation of nucleosomes. In contrast, in the *E. coli* nucleoid, about half of the chromosomal DNA is organized in the form of free, plectonemic supercoils (Fig 5) [90–92]. The remaining DNA is restrained in either the plectonemic form or alternative forms (see section 5.3.3), including but not limited to the toroidal form, by interaction with proteins such as NAPs. Thus, plectonemic supercoils represent effective supercoiling of the *E*. *coli* genome that is responsible for its condensation and organization. Both plectonemic and toroidal supercoiling aid in DNA condensation. It is noteworthy that because of the branching of plectonemic structures, it provides less DNA condensation than does the toroidal structure. The same size DNA molecule with equal supercoiling densities is more compact in a toroidal form than in a plectonemic form. In addition to condensing DNA, supercoiling aids in DNA organization. It promotes DNA disentanglement by reducing the probability of catenation [93]. Supercoiling also helps bring two distant sites of DNA in proximity thereby promoting a potential functional interaction between different segments of DNA.

#### Sources of supercoiling in *E*. *coli*

Three factors contribute to generating and maintaining chromosomal DNA supercoiling in *E. coli*: (i) activities of topoisomerases, (ii) the act of transcription, and (iii) NAPs.

#### Topoisomerases

Topoisomerases are a particular category of DNA metabolic enzymes that create or remove supercoiling by breaking and then re-ligating DNA strands [94]. *E*. *coli* possesses four topoisomerases (Table 3). DNA gyrase introduces negative supercoiling in the presence of ATP and removes positive supercoiling in the absence of ATP [95]. Across all forms of life, DNA gyrase is the only topoisomerase that can create negative supercoiling and it is because of this unique ability that bacterial genomes possess free negative supercoils; DNA gyrase is found in all bacteria but absent from higher eukaryotes. In contrast, Topo I opposes DNA gyrase by relaxing the negatively supercoiled DNA [96, 97]. There is genetic evidence to suggest that a balance between the opposing activities of DNA gyrase and Topo I are responsible for maintaining a steady-state level of average negative superhelicity in *E*. *coli* [98]. Both enzymes are essential for *E*. *coli* survival. A null strain of *topA*, the gene encoding Topo I, survives only because of the presence of suppressor mutations in the genes encoding DNA gyrase. These mutations result in reduced gyrase activity, suggesting that excess negative supercoiling due to the absence of Topo I is compensated by reduced negative supercoiling activity of DNA gyrase. Topo III is dispensable in *E*. *coli* and is not known to have any role in supercoiling in *E*. *coli* [99]. The primary function of Topo IV is to resolve sister chromosomes. However, it has been shown to also contribute to the steady-state level of negative supercoiling by relaxing negative supercoiling together with Topo I [100, 101].

**Table 3:** *E*. *coli* DNA topoisomerases.

Topoisomerase	Type	Function	Single- or double-stranded (SS or DS) cleavage
Topoisomerase I	IA	Removes (-) supercoiling	SS
Topoisomerase III	IA	Removes (-) supercoiling	SS
Topoisomerase IV	IIA	Removes (-) supercoiling	DS
DNA gyrase	IIA	Creates (-) supercoiling and removes (+) supercoiling	DS

#### Transcription

**Fig 6:**
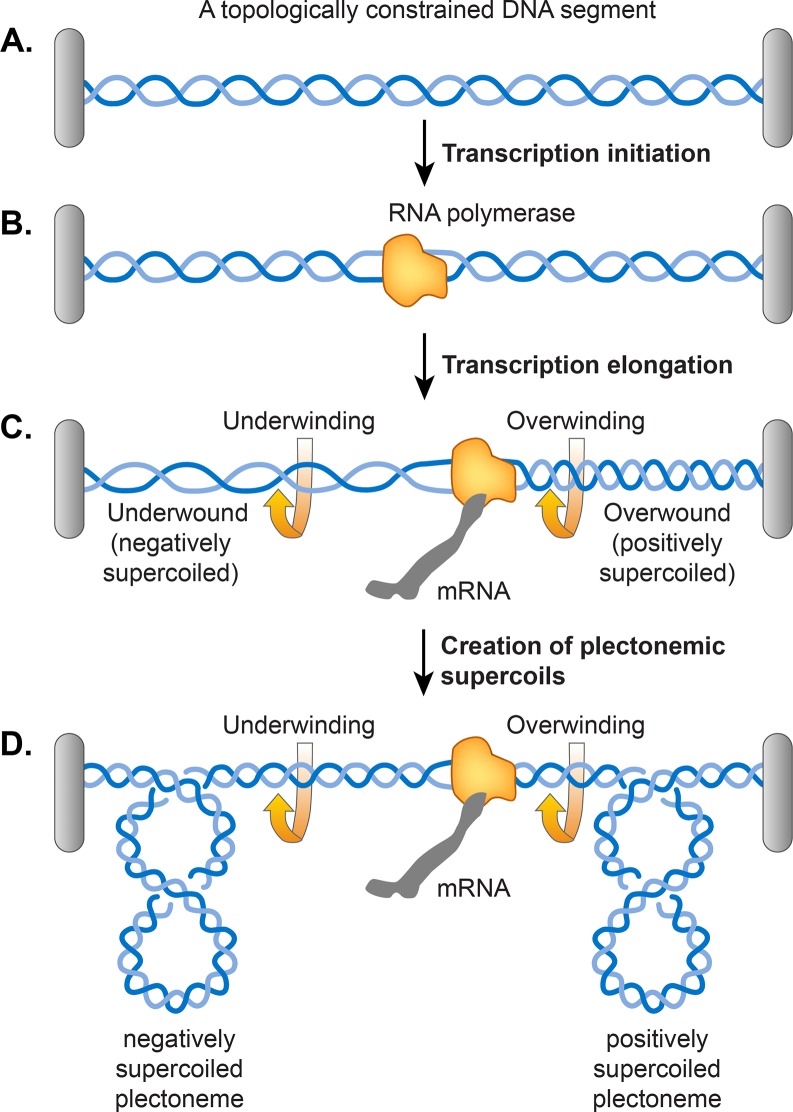
Twin supercoiling domain model for transcription-induced supercoiling. **A.** An example of topologically constrained DNA. A grey bar represents a topological constraint, e.g. a protein or a membrane anchor. **B.** Accommodation of RNA polymerase for transcription initiation results in the opening of the DNA double helix. **C.** An elongating RNA polymerase complex cannot rotate around the helical axis of DNA. Therefore, removal of helical turns by RNA polymerase causes overwinding of the topologically constrained DNA ahead and underwinding of the DNA behind, generating positively and negatively supercoiled DNA, respectively. Supercoiling can manifest as either change in the numbers of twists as shown in C or plectonemic writhe as shown in **D**.

A twin supercoiling domain model proposed by Liu and Wang argued that unwinding of DNA double helix during transcription induces supercoiling in DNA as shown in Fig 6 [102]. According to their model, transcribing RNA polymerase (RNAP) sliding along DNA forces the DNA to rotate on its helical axis. A hindrance in the free rotation of DNA might arise due to a topological constraint, causing the DNA in front of RNAP to become over-twisted (positively supercoiled) and the DNA behind RNAP would become under-twisted (negatively supercoiled) (Fig 6). It has been found that a topological constraint is not needed because RNAP generates sufficient torque that causes supercoiling even in a linear DNA template [103]. If DNA is already negatively supercoiled, this action relaxes existing negative supercoils before causing a buildup of positive supercoils ahead of RNAP and introduces more negative supercoils behind RNAP. In principle, DNA gyrase and Topo I should remove excess positive and negative supercoils respectively but if the RNAP elongation rate exceeds the turnover of the two enzymes, transcription contributes to the steady-state level of supercoiling.

#### Control of supercoiling by NAPs

In the eukaryotic chromatin, DNA is rarely present in the free supercoiled form because nucleosomes restrain almost all negative supercoiling through the tight binding of DNA to histones. Similarly, in *E*. *coli*, nucleoprotein complexes formed by NAPs restrain half of the supercoiling density of the nucleoid [89, 92]. In other words, if a NAP dissociates from a nucleoprotein complex, the DNA would adopt the free, plectonemic form. DNA binding of HU, Fis, and H-NS has been experimentally shown to restrain negative supercoiling in a relaxed but topologically constrained DNA [104–108]. They can do so either by changing the helical pitch of DNA or generating toroidal writhes by DNA bending and wrapping (Fig 2). Alternatively, NAPs can preferentially bind to and stabilize other forms of the underwound DNA such as cruciform structures and branched plectonemes. Fis has been reported to organize branched plectonemes through its binding to cross-over regions (Fig 5) and HU preferentially binds to cruciform structures [108].

NAPs also regulate DNA supercoiling indirectly. Fis can modulate supercoiling by repressing the transcription of the genes encoding DNA gyrase [109]. There is genetic evidence to suggest that HU controls supercoiling levels by stimulating DNA gyrase and reducing the activity of Topo I [110, 111]. In support of the genetic studies, HU was shown to stimulate DNA gyrase-catalyzed decatenation of DNA *in vitro* [112]. It is unclear mechanistically how HU modulates the activities of the gyrase and Topo I. HU might physically interact with DNA gyrase and Topo I or DNA organization activities of HU such as DNA bending may facilitate or inhibit the action of DNA gyrase and Topo I respectively.

#### Plectonemic supercoils organize into multiple topological domains

**Fig 7:**
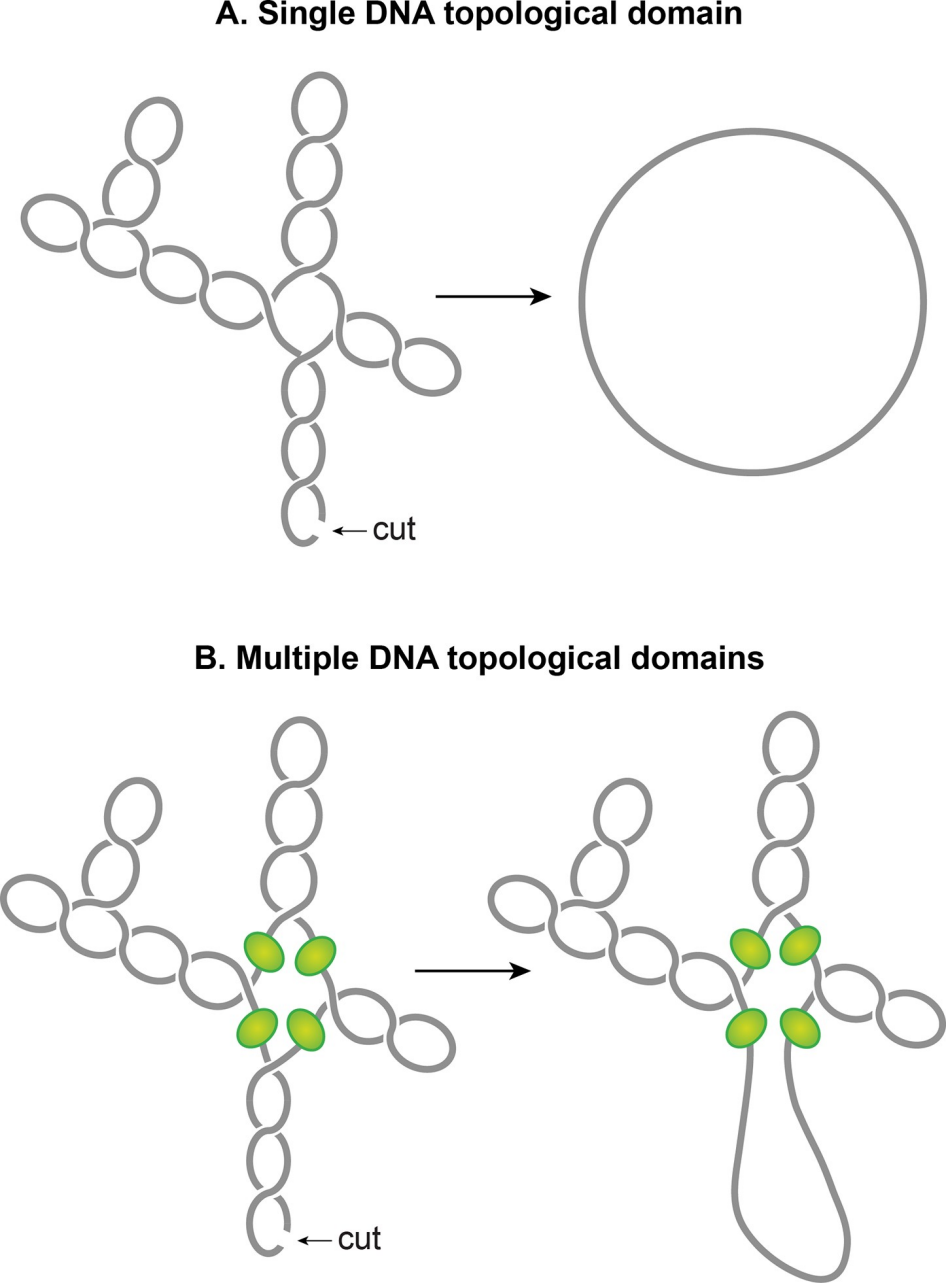
The chromosomal DNA within the nucleoid is segregated into independent supercoiled topological domains. **A.** An illustration of a single topological domain of a supercoiled DNA. A single double-stranded cut anywhere would be sufficient to relax the supercoiling tension of the entire domain. **B.** An illustration of multiple topological domains in a supercoiled DNA molecule. A presence of supercoiling-diffusion barriers segregates a supercoiled DNA molecule into multiple topological domains. Hypothetical supercoiling diffusion barriers are represented as green spheres. As a result, a single double-stranded cut will only relax one topological domain and not the others. Plectonemic supercoils of DNA within the *E*. *coli* nucleoid are organized into several topological domains, but only four domains with a different number of supercoils are shown for simplicity.

One of the striking features of the nucleoid is that plectonemic supercoils are organized into multiple topological domains (Fig 7) [113]. In other words, a single cut in one domain will only relax that domain and not the others. A topological domain forms because of a supercoiling-diffusion barrier. Independent studies employing different methods have reported that the topological domains are variable in size ranging from 10–400 kb [91, 113, 114]. Random placement of barriers commonly observed in these studies seems to explain the wide variability in the size of domains.

Although identities of domain barriers remain to be established, possible mechanisms responsible for the formation of the barriers include: (i) A domain barrier could form when a protein with an ability to restrain supercoils simultaneously binds to two distinct sites on the chromosome forming a topologically isolated DNA loop or domain. It has been experimentally demonstrated that protein-mediated looping in supercoiled DNA can create a topological domain [115, 116]. NAPs such as H-NS and Fis are potential candidates, based on their DNA looping abilities and the distribution of their binding sites. (ii) Bacterial interspersed mosaic elements (BIMEs) also appear as potential candidates for domain barriers. BIMEs are palindromic repeats sequences that are usually found between genes. A BIME has been shown to impede supercoiling diffusion in a synthetically designed topological cassette inserted in the *E*. *coli* chromosome [117]. There are ~600 BIMEs distributed across the genome, possibly dividing the chromosome into 600 topological domains [118]. (iii) Barriers could also result from the attachment of DNA to the cell membrane through a protein that binds to both DNA and membrane or through nascent transcription and the translation of membrane-anchored proteins (see section 5.2). (iv) Transcription activity can generate supercoiling-diffusion barriers. An actively transcribing RNAP has been shown to block the dissipation of plectonemic supercoils, thereby forming a supercoiling-diffusion barrier [119–121].

### Spatial organization of the nucleoid

#### Chromosomal interaction domains

**Fig 8:**
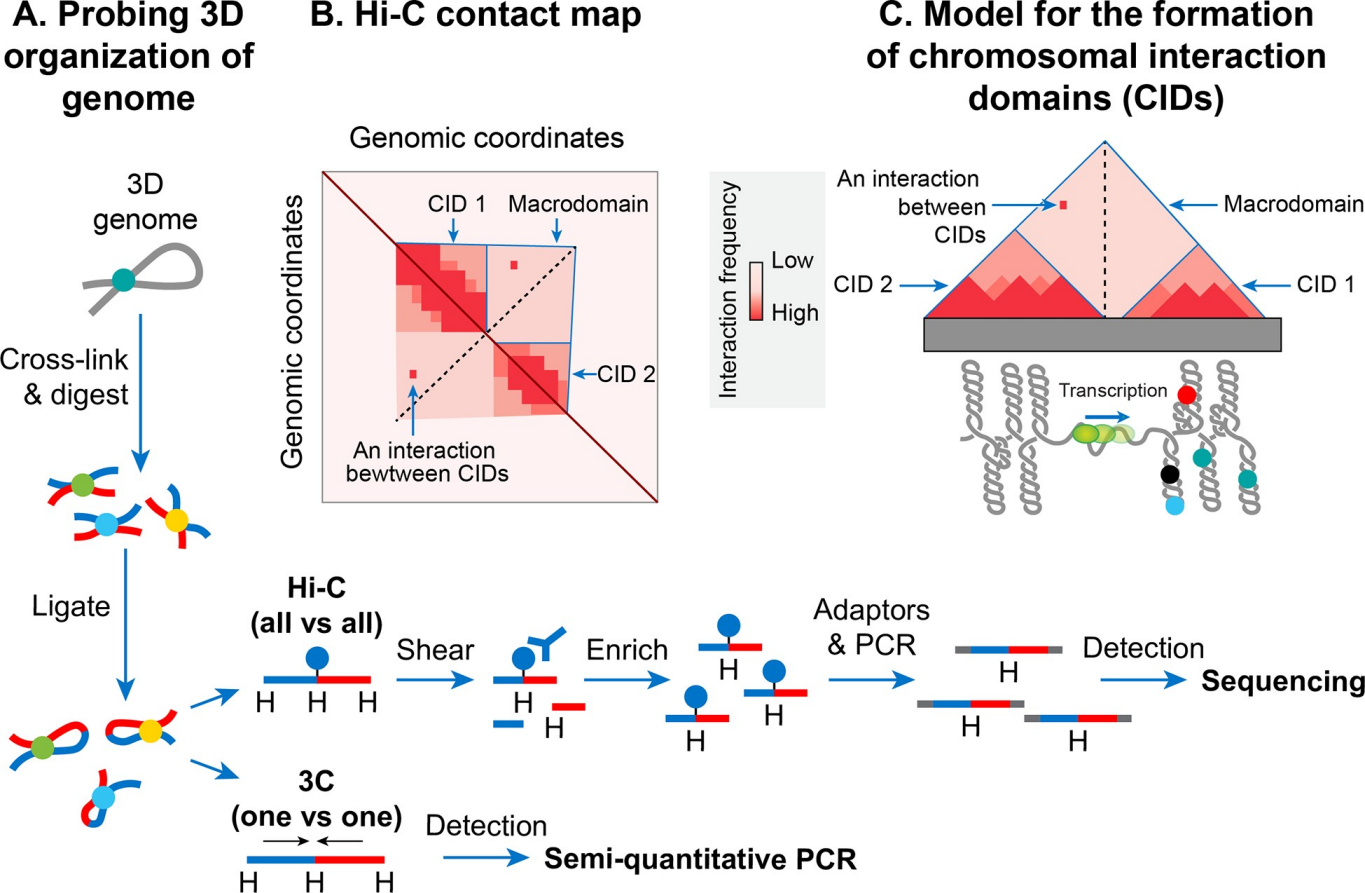
Nucleoid is spatially organized into chromosomal interactions domains (CIDs) and macrodomains. **A.** Chromosome conformation capture (3C) methods probe 3D genome organization by quantifying physical interactions between genomic loci that are nearby in 3D-space but may be far away in the linear genome. A genome is cross-linked with formaldehyde to preserve physical contacts between genomic loci. Subsequently, the genome is digested with a restriction enzyme. In the next step, a DNA ligation is carried out under diluted DNA concentrations to favor intra-molecular ligation (between cross-linked fragments that are brought into physical proximity by 3D genome organization). A frequency of ligation events between distant DNA sites reflects a physical interaction. In the 3C method, ligation junctions are detected by the semi-quantitative PCR amplification in which amplification efficiency is a rough estimate of pairwise physical contact between genomic regions of interests and its frequency. The 3C method probes a physical interaction between two specific regions identified a priori, whereas its Hi-C version detects physical interactions between all possible pairs of genomic regions simultaneously. In the Hi-C method, digested ends are filled in with a biotinylated adaptor before ligation. Ligated fragments are sheared and then enriched by a biotin-pull down. Ligation junctions are then detected and quantified by the paired-end next-generation sequencing methods. **B.** Hi-C data are typically represented in the form of a two-dimensional matrix in which the x-axis and y-axis represent the genomic coordinates. The genome is usually divided into bins of a fixed size, e.g., 5-kb. The size of bins essentially defines the contact resolution. Each entry in the matrix, m_ij_, represents the number of chimeric sequencing reads mapped to genomic loci in bins i and j. A quantification of the reads (represented as a heatmap) denotes the relative frequency of contacts between genomic loci of bins i and j. A prominent feature of the heatmap is a diagonal line that appears due to more frequent physical interaction between loci that are very close to each other in the linear genome. The intensity as we move away from this diagonal line represents the relative frequency of physical interaction between loci that are far away from each other in the linear genome. Triangles of high-intensity along the diagonal line represent highly self-interacting chromosomal interaction domains (CIDs) that are separated by a boundary region that consists of a smaller number of interactions. **C.** In many bacterial species including *E*. *coli*, it appears that supercoiled topological domains organize as CIDs. Plectonemic supercoiling promotes a high level of interaction among genomic loci within a CID, and a plectoneme-free region (PFR), created due to high transcription activity, acts as a CID boundary. Nucleoid-associated proteins, depicted as closed circles, stabilize the supercoiling-mediated interactions. The actively transcribing RNA polymerase (depicted as a green sphere) in the PFR blocks dissipation of supercoiling between the two domains thus acts as a supercoiling diffusion barrier. The size of the CIDs ranges between 30–400 kb. Several triangles (CIDs) merge to form a bigger triangle that represents a macrodomain. In other words, CIDs of a macrodomain physically interact with each other more frequently than with CIDs of a neighboring macrodomain or with genomic loci outside of that macrodomain. A macrodomain may comprise of several CIDs. For simplicity, a macrodomain comprising of only two CIDs is shown.

In recent years, the advent of a molecular method called chromosome conformation capture (3C) has allowed studying a high-resolution spatial organization of chromosomes in both bacteria and eukaryotes [122]. 3C and its version that is coupled with deep sequencing (Hi-C) [123] determine physical proximity, if any, between any two genomic loci in 3D space (Fig 8A and 8B). A high-resolution contact map of bacterial chromosomes including the *E*. *coli* chromosome has revealed that a bacterial chromosome is segmented into many highly self-interacting regions called chromosomal interaction domains (CIDs) (Fig 8B) [124–126]. CIDs are equivalent to topologically associating domains (TADs) observed in many eukaryotic chromosomes [127], suggesting that the formation of CIDs is a general phenomenon of genome organization. Two characteristics define CIDs or TADs. First, genomic regions of a CID physically interact with each other more frequently than with the genomic regions outside that CID or with those of a neighboring CID. Second, the presence of a boundary between CIDs that prevents physical interactions between genomic regions of two neighboring CIDs.

The *E*. *coli* chromosome was found to consist of 31 CIDs in the growth phase [124]. The size of the CIDs ranged from 40 to ~300 kb. It appears that a supercoiling-diffusion barrier responsible for segregating plectonemic DNA loops into topological domains functions as a CID boundary in *E*. *coli* and many other bacteria. In other words, the presence of a supercoiling-diffusion barrier defines the formation of CIDs. Findings from the Hi-C probing of chromosomes in *E*. *coli* [124], *Caulobacter crescentus* [125], and *Bacillus subtilis* [126] converge on a model that CIDs form because plectonemic looping together with DNA organization activities of NAPs promotes physical interactions among genomic loci, and a CID boundary consists of a plectoneme-free region (PFR) that prevents these interactions (Fig 8C). A PFR is created due to high transcription activity because the helical unwinding of DNA by actively transcribing RNAP restrains plectonemic supercoils. As a result, supercoil dissipation is also blocked, creating a supercoiling-diffusion barrier. Indirect evidence for this model comes from an observation that CIDs of bacterial chromosomes including the *E*. *coli* chromosome display highly transcribed genes at their boundaries, indicating a role of transcription in the formation of a CID boundary [124, 125]. More direct evidence came from a finding that the placement of a highly transcribed gene at a position where no boundary was present created a new CID boundary in the *C*. *crescentus* chromosome [125]. However, not all CID boundaries correlated with highly transcribed genes in the *E*. *coli* chromosome suggesting that other unknown factors are also responsible for the formation of CID boundaries and supercoiling diffusion barriers.

#### Macrodomains

Plectonemic DNA loops organized as topological domains or CIDs appear to coalesce further to form large spatially distinct domains called macrodomains [128, 129]. In *E*. *coli*, macrodomains were initially identified as large segments of the genome whose DNA markers localized together (co-localized) in fluorescence in situ hybridization (FISH) studies. A large genomic region (~1-Mb) covering *oriC* (origin of chromosome replication) locus co-localized and was called Ori macrodomain. Likewise, a large genomic region (~1-Mb) covering the replication terminus region (*ter*) co-localized and was called Ter macrodomain. Macrodomains were later identified based on how frequently pairs of lambda *att* sites that were inserted at various distant locations in the chromosome recombined with each other. In this recombination-based method, a macrodomain was defined as a large genomic region whose DNA sites can primarily recombine with each other, but not with outside of that macrodomain. The recombination-based method confirmed the Ori and Ter macrodomains that were identified in FISH studies and identified two additional macrodomains [7, 130].

The two additional macrodomains were formed by the additional ~1-Mb regions flanking the Ter and were referred to as Left and Right. These four macrodomains (Ori, Ter, Left, and Right) comprised most of the genome, except for two genomic regions flanking the Ori (Fig 1). These two regions (NS-L and NS-R) were more flexible and non-structured compared to a macrodomain as DNA sites in them recombined with DNA sites located in macrodomains on both sides. The genetic position of *oriC* appears to dictate the formation of macrodomains, because repositioning of *oriC* by genetic manipulation results in the reorganization of macrodomains. For example, genomic regions closest to the *oriC* always behave as an NS regardless of DNA sequence and regions further away behave as macrodomains [131].

The Hi-C technique further confirmed a hierarchical spatial organization of CIDs in the form of macrodomains [124]. In other words, CIDs of a macrodomain physically interacted with each other more frequently than with CIDs of a neighboring macrodomain or with genomic loci outside of that macrodomain (Fig 8B and 8C). The Hi-C data showed that the *E*. *coli* chromosome was partitioning into two distinct domains. The region surrounding *ter* formed an insulated domain that overlapped with the previously identified Ter macrodomain. DNA-DNA contacts in this domain occurred only in the range of up to ~280 kb. The rest of the chromosome formed a single domain whose genomic loci exhibited contacts in the range of >280-kb. While most of the contacts in this domain were restricted to a maximum distance of ~500 kb, there were two loose regions whose genomic loci formed contacts at even greater distances (up to ~1 Mb). These loose regions corresponded to the previously identified flexible and less-structured regions (NS). The boundaries of the insulated domain encompassing *ter* and the two loose regions identified by the Hi-C method segmented the entire chromosome into six regions that correspond with the four macrodomains and two NS regions defined by recombination-based assays. Thus, the two approaches were in good agreement with one another.

### Proteins that drive macrodomain formation

#### MatP

**Fig 9:**
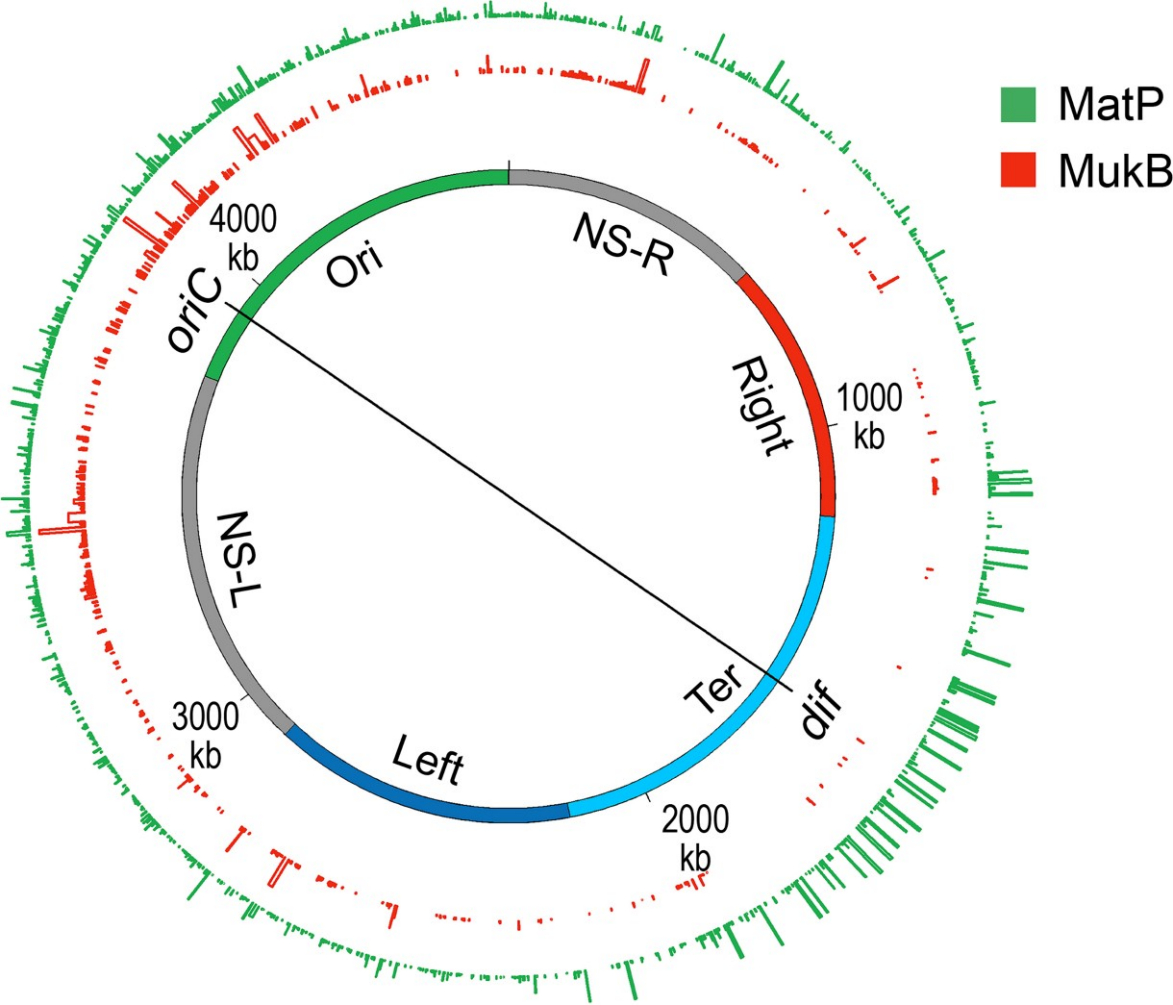
Genome-wide occupancy of MatP and MukB of *E*. *coli*. A circular layout of the *E*. *coli* genome depicting genome-wide occupancy of MatP and MukB in *E*. *coli*. The innermost circle depicts the *E*. *coli* genome. The regions of the genome which organize as spatial domains (macrodomains) in the nucleoid are indicated as colored bands. The genome occupancy of each protein, determined by ChIP-Seq, is plotted as a histogram in outside circles (bin size 300 bp) in which the bar height is indicative of relative binding enrichment. The figure was prepared in circos/0.69–6 using the processed ChIP-Seq data from [132].

A search for protein(s) responsible for macrodomain formation led to the identification of Macrodomain Ter protein (MatP) [27]. MatP almost exclusively binds in the Ter macrodomain by recognizing a 13-bp motif called the macrodomain *ter* sequence (*matS*) (Table 2). There are 23 *matS* sites present in the Ter macrodomain, on average there is one site every 35-kb. Further evidence of MatP binding in the Ter macrodomain comes from fluorescence imaging of MatP. Discrete MatP foci were observed that co-localized with Ter macrodomain DNA markers [27]. A strong enrichment of ChIP-Seq signal in the Ter macrodomain also corroborates the preferential binding of MatP to this macrodomain (Fig 9).

**Fig 10:**
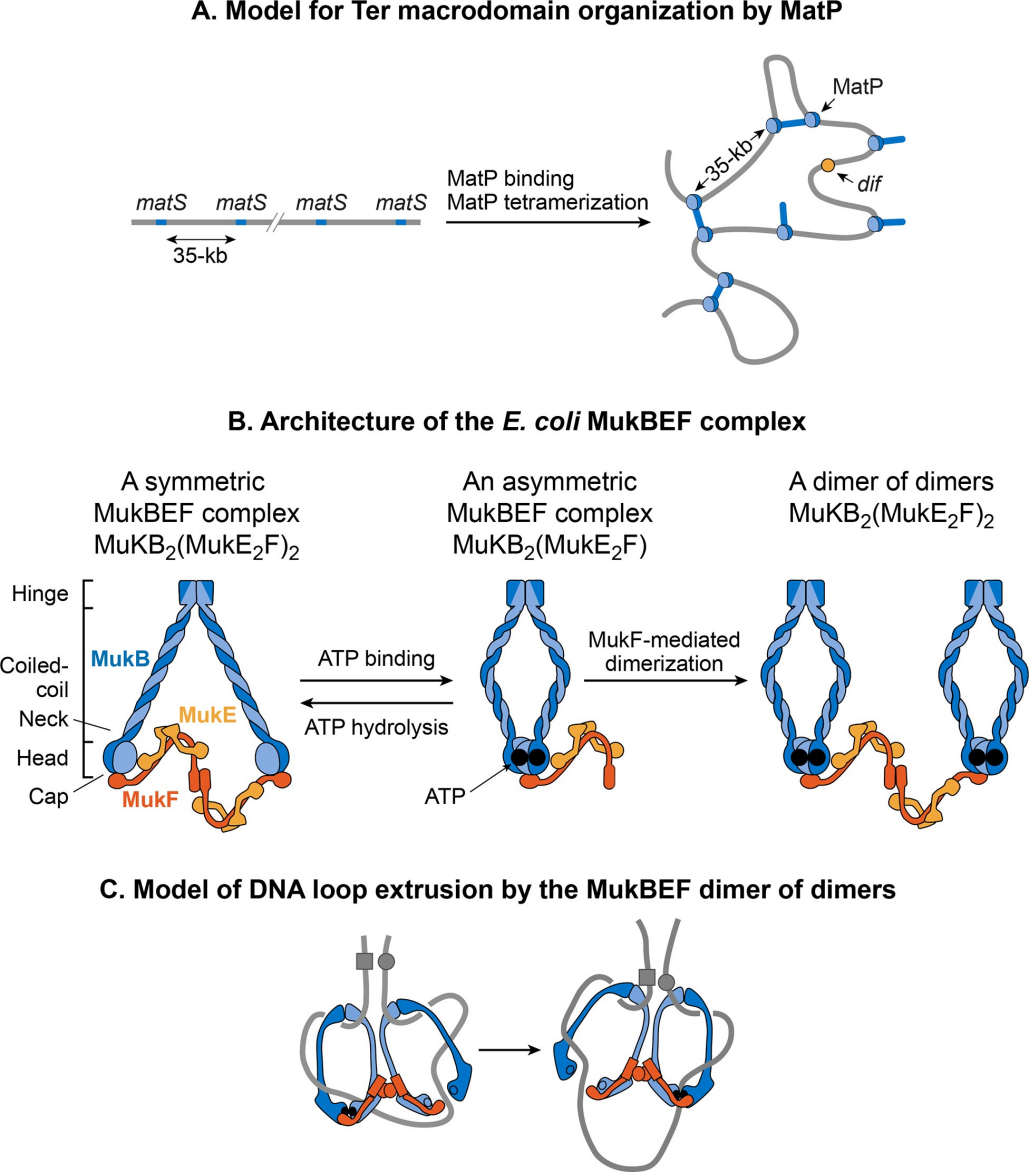
Models for DNA organization by MatP and MukBEF. **A.** A *matS*-bridging model for DNA organization in the Ter macrodomain by MatP. MatP recognizes a 13-bp signature DNA sequence called *matS* that is present exclusively in the Ter macrodomain. There are 23 *matS* sites separated by one another by an average of 35-kb. MatP binds to a *matS* site as a dimer, and the tetramerization of the DNA-bound dimers bridges *matS* sites forming large DNA loops. **B.** The architecture of the *E*. *coli* MukBEF complex. The complex is formed by protein-protein interactions between MukB (blue), MukF (dark orange) and MukE (light orange). MukB, which belongs to the family of structural maintenance of chromosomes (SMCs) proteins, forms a dimer (monomers are shown by dark and light blue colors) consisting of an ATPase head domain and a 100 nm long intramolecular coiled-coil with a hinge region in the middle. Because of the flexibility of the hinge region, MukB adopts a characteristic V-shape of the SMC family. MukF also tends to exist as a dimer because of the strong dimerization affinity between monomers [134, 135]. The C-terminal domain of MukF can interact with the head domain of MukB while its central domain can interact with MukE. Two molecules of MukE and one molecule of MukF associate with each other independent of MukB to form a trimeric complex (MukE_2_F). Since MukF tends to exist in a dimeric form, the dimerization of MukF results in an elongated hexameric complex (MukE_2_F)_2_ [134]. In the absence of ATP, the (MukE_2_F)_2_ complex binds to the MukB head domains through the C-terminal domain of MukF to form a symmetric MukBEF complex (shown on the left). The stoichiometry of the symmetric complex is B_2_(E_2_F)_2_. The ATP binding between the MukB head domains forces the detachment of one MukF molecule and two MukE molecules [134, 136]. As a result, an asymmetric MukBEF complex of the stoichiometry B_2_(E_2_F)_1_ is formed. Since MukF readily dimerizes, the MukF dimerization can potentially join two ATP-bound asymmetric molecules resulting in the formation of a dimer of dimers with the stoichiometry of B_4_(E_2_F)_2_ (shown on the right). The stoichiometry of the MukBEF complex in vivo is estimated to be B_4_(E_2_F)_2_ suggesting that a dimer of dimers is the functional unit in vivo [137]. **C.** A model for loop extrusion by a MukBEF dimer of dimers. A dimer of dimer loads onto DNA (depicted as a grey line) through DNA binding domains of MukB. MukB has been shown to bind DNA via its hinge region and the top region of its head domain [134, 138]. The translocation of the complex away from its loading site then extrudes DNA loops. The loops are extruded in a rock-climbing manner by the coordinated opening and closing of the MukBEF ring through the MukB head disengagement that occurs due to coordinated ATP hydrolysis in the two dimers [137]. Dark and light blue circles represent ATP binding and hydrolysis events respectively. MukE is not shown in the complex for simplicity.

MatP condenses DNA in the Ter macrodomain because the lack of MatP increased the distance between two fluorescent DNA markers located 100-kb apart in the Ter macrodomain. Furthermore, MatP is a critical player in insulating the Ter macrodomain from the rest of the chromosome [124]. It promotes DNA-DNA contacts within the Ter macrodomain but prevents contacts between the DNA loci of Ter domain and those of flanking regions. How does MatP condense DNA and promote DNA-DNA contacts? The experimental results are conflicting. MatP can form a DNA loop between two *matS* sites in vitro and its DNA looping activity depends on MatP tetramerization. Tetramerization occurs via coiled-coil interactions between two MatP molecules bound to DNA [133]. One obvious model based on in vitro results is that MatP promotes DNA-DNA contacts in vivo by bridging *matS* sites (Fig 10A). However, although MatP connected distant sites in Hi-C studies, it did not specifically connect the *matS* sites [124]. Furthermore, a MatP mutant that was unable to form tetramers behaved like wild-type. These results argue against the *matS* bridging model for Ter organization, leaving the mechanism of MatP action elusive. One possibility is that MatP spreads to nearby DNA segments from its primary *matS* binding site and bridge distant sites via a mechanism that does not depend on the tetramerization.

#### MukBEF

MukB belongs to a family of ATPases called structural maintenance of chromosome proteins (SMCs), which participate in higher-order chromosome organization in eukaryotes [139]. Two MukB monomers associate via continuous antiparallel coiled-coil interaction forming a 100-nm long rigid rod. A flexible hinge region occurs in the middle of the rod [140, 141]. Due to the flexibility of the hinge region, MukB adopts a characteristic V-shape of the SMC family (Fig 10B). The non-SMC subunits associating with MukB are MukE and MukF. The association closes the V formation, resulting in large ring-like structures (Fig 10B). MukE and MukF are encoded together with MukB in the same operon in *E*. *coli* [142]. The deletion of either subunit results in the same phenotype suggesting that the MukBEF complex is the functional unit in vivo [137]. DNA binding activities of the complex reside in the MukB subunit, whereas MukE and MukF modulate MukB activity.

MukBEF complex, together with Topo IV, is required for decatenation and repositioning of newly replicated *oriC*s [132, 143–146] The role of MukBEF is not restricted during DNA replication [147]. It organizes and condenses DNA even in non-replicating cells. The recent high-resolution chromosome conformation map of the MukB-depleted *E*. *coli* strain reveals that MukB participates in the formation of DNA-DNA interactions on the entire chromosome, except in the Ter macrodomain [124]. How is MukB prevented from acting in the Ter macrodomain? MatP physically interacts with MukB, thus preventing MukB from localizing to the Ter macrodomain [132]. This is evident in the DNA binding of MatP and MukB in the Ter macrodomain. MatP DNA binding is enriched in the Ter macrodomain, whereas MukB DNA binding is reduced compared to the rest of the genome (Fig 9). Furthermore, in a strain already lacking MatP, the absence of MukB causes a reduction in DNA contacts throughout the chromosome, including the Ter macrodomain [124]. This result agrees with the view that MatP displaces MukB from the Ter domain.

How does the MukBEF complex function to organize the *E*. *coli* chromosome? According to the current view, SMC complexes organize chromosomes by extruding DNA loops [148]. SMC complexes translocate along DNA to extrude loops in a cis-manner (on the same DNA molecule), wherein the size of loops depends on the processivity of the complex. SMC complexes from different organisms differ in the mechanism of loop extrusion [148]. Single molecule fluorescence microscopy of MukBEF in *E*. *coli* suggests that the minimum functional unit in vivo is a dimer of dimers (Fig 10B) [137]. This unit is formed by joining of two ATP-bound MukBEF complexes through MukF-mediated dimerization. MukBEF localizes in the cell as 1–3 clusters that are elongated parallel to the long axis of the cell. Each cluster contains an average ~ 8–10 dimers of dimers. According to the current model, the MukBEF extrudes DNA loops in a “rock-climbing” manner (Fig 10C) [137, 149]. A dimer of the dimers releases one segment of DNA and capture a new DNA segment without dissociating from the chromosome. Besides DNA looping, a link between negative supercoiling and in vivo MukBEF function together with the ability of the MukB subunit to constrain negative supercoils in vitro suggests that MukBEF organizes DNA by generating supercoils [150–152]. A full understanding of a molecular mechanism of MukBEF action in vivo warrants further investigations.

#### Spatial organization of the nucleoid by NAPs and naRNAs

In addition to contributing to the chromosome compaction by bending, bridging, and looping DNA at a smaller scale (~1-kb), NAPs participate in DNA condensation and organization by promoting long-rang DNA-DNA contacts. Two NAPs, Fis and HU, emerged as the key players in promoting long-range DNA-DNA contacts that occur throughout the chromosome [124]. It remains to be studied how DNA organization activities of Fis and HU that are well understood at a smaller scale (~1-kb) results in the formation of long-range DNA-DNA interactions. Nonetheless, some of the HU-mediated DNA interactions require the presence of naRNA4 [84]. naRNA4 also participates in making long-range DNA contacts. HU catalyzes some of the contacts, not all, suggesting that RNA participates with other NAPs in forming DNA contacts. HU also appears to act together with MukB to promote long-range DNA-DNA interactions. This view is based on observations that the absence of either HU or MukB caused a reduction in the same DNA-DNA contacts [124]. It is unclear how MukB and HU potentially act together in promoting DNA-DNA interactions. The two proteins may interact physically. Alternatively, while MukBEF extrudes large DNA loops, HU condenses and organizes those loops by mechanisms described in section 2.1.1.

#### Spatial organization of the nucleoid by functional relatedness of genes

There are reports that functionally-related genes of *E*. *coli* are physically together in 3-D space within the chromosome even though they are far apart by genetic distance. Spatial proximity of functionally-related genes not only makes the biological functions more compartmentalized and efficient but would also contribute to the folding and spatial organization of the nucleoid. A recent study using fluorescent markers for detection of specific DNA loci examined pairwise physical distances between the seven rRNA operons that are genetically separated from each other (by as much as two million bp). It reported that all of the operons, except *rrn*C, were in physical proximity [153, 154]. Surprisingly, 3C-seq studies did not reveal the physical clustering of *rrn* operons [124], contradicting the results of the fluorescence-based study. Therefore, further investigation is required to resolve these contradicting observations. In another example, GalR forms an interaction network of GalR binding sites that are scattered across the chromosome [155]. GalR is a transcriptional regulator of the galactose regulon comprised of genes encoding enzymes for transport and metabolism of the sugar D-galactose [156]. GalR exists in only one to two foci in cells [155] and can self-assemble into large ordered structures [157]. Therefore, it appears that DNA-bound GalR multimerizes to form long-distance interactions.

### Nucleoid global shape and structure

#### The nucleoid is a helical ellipsoid, radially confined in the cell

**Fig 11:**
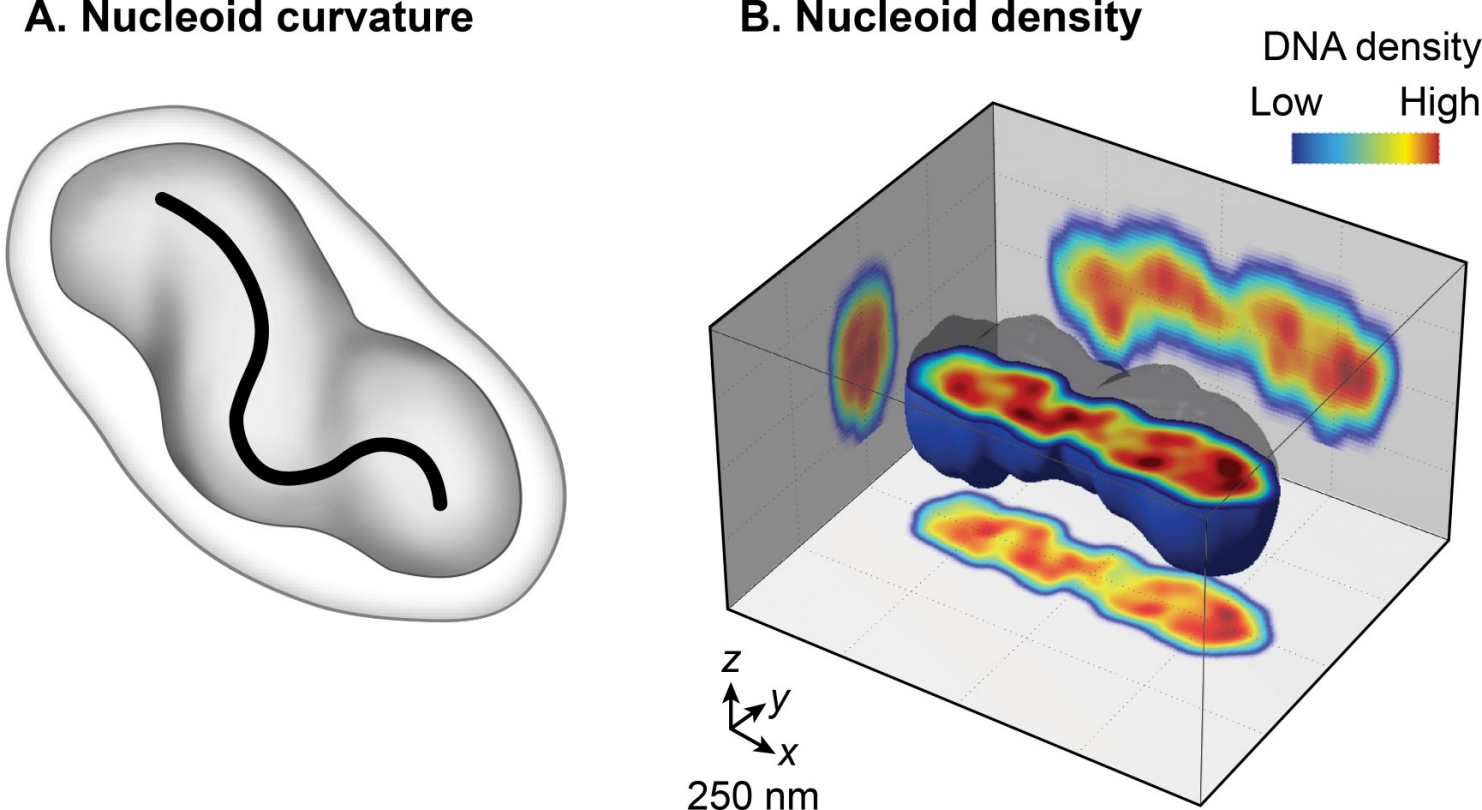
Nucleoid as a helical ellipsoid with longitudinal high-density DNA regions. **A.** A cartoon of *E*. *coli* cell with a curved nucleoid (dark grey). A curved centroids path, denoted by black line, emphasizes the curved shape of the nucleoid. The cartoon is based on a figure in [8]. **B.** Cross-sectioning of the *E*. *coli* nucleoid visualized by HU-mCherry. Fluorescence intensity is taken as a proxy for DNA density and is represented by blue to red in increasing order. Data were taken from [9].

Conventional transmission electron microscopy (TEM) of chemically fixed *E*. *coli* cells portrayed the nucleoid as an irregularly shaped organelle. However, wide-field fluorescence imaging of live nucleoids in 3D revealed a discrete, ellipsoid shape (Fig 11) [4, 8–10]. The overlay of a phase-contrast image of the cell and the fluorescent image of the nucleoid showed a close juxtaposition only in the radial dimension along its entire length of the nucleoid to the cell periphery. This finding indicates the radial confinement of the nucleoid [8]. A detailed examination of the 3D fluorescence image after cross-sectioning perpendicular to its long axis further revealed two global features of the nucleoid: curvature and longitudinal, high-density regions. Examining the chirality of the centerline of the nucleoid by connecting the center of intensity of each cross-section showed that the overall nucleoid shape is curved (Fig 11A) [158]. The fluorescence intensity distribution in the cross-sections revealed a density substructure, consisting of curved, high-density regions or bundles at the central core, and low-density regions at the periphery (Fig 11B) [8, 9]. One implication of the radial confinement is that it determines the curved shape of the nucleoid. According to one model, the nucleoid is forced to bend because it is confined into a cylindrical *E*. *coli* cell whose radius is smaller than its bendable length (persistence length) [8]. This model was supported by observations that removal of the cell wall or inhibition of cell wall synthesis increased the radius of the cell and resulted in a concomitant increase in the helical radius and a decrease in the helical pitch in the nucleoid [8].

#### Connections between nucleoid and cell membrane

An expansion force due to connections between DNA within the nucleoid and cell membrane (DNA-membrane connections) appears to function in opposition to condensation forces to maintain an optimal condensation level of the nucleoid. Cell-fractionation and electron microscopy studies first indicated the possibility of DNA-membrane connections [159, 160]. There are now several known examples of DNA-membrane connections. Transertion is a mechanism of concurrent transcription, translation, and insertion of nascent membrane proteins that forms transient DNA-membrane contacts [161]. Transertion of two membrane proteins, LacY and TetA, has been demonstrated to cause the repositioning of chromosomal loci toward the membrane [162]. Another mechanism of nucleoid-membrane connections is through a direct contact between membrane-anchored transcription regulators and their target sites in the chromosome. One example of such as transcription regulator in *E*. *coli* is CadC. CadC contains a periplasmic sensory domain and a cytoplasmic DNA binding domain. Sensing of an acidic environment by its periplasmic sensory domain stimulates the DNA binding activity of CadC, which then activates transcription of its target genes [163]. The membrane-localization of genes regulated by a membrane-anchored transcription regulator is yet to be demonstrated. Nonetheless, activation of target genes in the chromosome by these regulators is expected to result in a nucleoid-membrane contact albeit it would be a dynamic contact. Besides these examples, the chromosome is also specifically anchored to the cell membrane through protein-protein interaction between DNA-bound proteins, e.g., SlmA and MatP, and the divisome [164, 165].

Since membrane-protein encoding genes are distributed throughout the genome, dynamic DNA-membrane contacts through transertion can act as a nucleoid expansion force. This expansion force would function in opposition to condensation forces to maintain an optimal condensation level. The formation of highly condensed nucleoids upon the exposure of *E*. *coli* cells to chloramphenicol, which blocks translation, provides support for the expansion force of transient DNA-membrane contacts formed through transertion. The round shape of overly-condensed nucleoids after chloramphenicol treatment also suggests a role for transertion-mediated DNA-membrane contacts in defining the ellipsoid shape of the nucleoid.

### Growth-phase dependent nucleoid dynamics

The nucleoid reorganizes in stationary phase cells suggesting that the nucleoid structure is highly dynamic, determined by the physiological state of cells. A comparison of high-resolution contact maps of the nucleoid revealed that the long-range contacts in the Ter macrodomain increased in the stationary phase, compared to the growth phase [124]. Furthermore, CID boundaries in the stationary phase were different from those found in the growth phase. Finally, nucleoid morphology undergoes a massive transformation during prolonged stationary phase [166]; the nucleoid exhibits ordered, toroidal structures [167].

Growth-phase specific changes in nucleoid structure could be brought about by a change in levels of nucleoid-associated DNA architectural proteins (the NAPs and the Muk subunits), supercoiling, and transcription activity. The abundance of NAPs and the Muk subunits changes according to the bacterial growth cycle. Fis and the starvation-induced DNA binding protein Dps, another NAP, are almost exclusively present in the growth phase and stationary phase respectively. Fis levels rise upon entry into the exponential phase and then rapidly decline while cells are still in the exponential phase, reaching levels that are undetectable in the stationary phase [168]. While Fis levels start to decline, levels of Dps start to rise and reach a maximum in the stationary phase [16]. A dramatic transition in the nucleoid structure observed in the prolonged stationary phase has been mainly attributed to Dps. It forms DNA/ crystalline assemblies that act to protect the nucleoid from DNA damaging agents present during starvation [167].

HU, IHF, and H-NS are present in both the growth phase and the stationary phase [16]. However, their abundance changes significantly such that HU and Fis are the most abundant NAPs in the growth phase, whereas IHF and Dps become the most abundant NAPs in the stationary phase [16]. HUαα is the predominant form in the early exponential phase, whereas the heterodimeric form HUαß predominates in the stationary phase, with minor amounts of homodimers [169]. This transition has functional consequences regarding nucleoid structure because HUαα and HUαß appear to organize and condense DNA differently; both form filaments, but only HUαα can bring multiple DNA segments together (DNA bunching) to form a DNA network [40]. The copy number of MukB increases two-fold in the stationary phase [136, 147]. An increase in the number of MukB molecules could have an influence on the processivity of the MukBEF complex as a DNA loop extruding factor resulting in larger or a greater number of the loops.

Supercoiling can act in a concerted manner with DNA architectural proteins to reorganize the nucleoid. The overall supercoiling level decreases in the stationary phase, and supercoiling exhibits a different pattern at the regional level [170]. Changes in supercoiling can alter the topological organization of the nucleoid. Furthermore, because a chromosomal region of high transcription activity forms a CID boundary, changes in transcription activity during different growth phases could alter the formation of CID boundaries, and thus the spatial organization of the nucleoid. It is possible that changes in CID boundaries observed in the stationary phase could be due to the high expression of a different set of genes in the stationary phase compared to the growth phase [124].

### Nucleoid structure and gene expression

#### NAPs and gene expression

The *E*. *coli* chromosome structure and gene expression appear to influence each other reciprocally. On the one hand, a correlation of a CID boundary with high transcription activity indicates that chromosome organization is driven by transcription. On the other hand, the 3D structure of DNA within nucleoid at every scale may be linked to gene expression. First, it has been shown that a reorganization of the 3D architecture of the nucleoid in *E*. *coli* can dynamically modulate cellular transcription pattern [171]. A mutant of HUα made the nucleoid very much condensed by increased positive superhelicity of the chromosomal DNA. Consequently, many genes were repressed, and many quiescent genes were expressed. Besides, there are many specific cases in which protein-mediated local architectural changes (Fig 2) alter gene transcription. For example, the formation of rigid nucleoprotein filaments by H-NS blocks RNAP access to the promoter thus prevent gene transcription [172]. Through gene silencing, H-NS acts as a global repressor preferentially inhibiting transcription of horizontally transferred genes [20, 46]. In another example, the specific binding of HU at the *gal* operon facilitates the formation of a DNA loop that keeps the *gal* operon repressed in the absence of the inducer [173]. The topologically distinct DNA micro-loop created by coherent bending of DNA by Fis at stable RNA promoters activates transcription. DNA bending by IHF differentially controls transcription from the two tandem promoters of the *ilvGMEDA* operon in *E*. *coli* [174, 175]. It is noteworthy that specific topological changes by NAPs not only regulate gene transcription but are also involved in other processes such as DNA replication initiation, recombination, and transposition. In contrast to specific gene regulation, how higher-order chromosome structure and its dynamics influences gene expression globally at the molecular level remains to be worked out.

#### DNA supercoiling and gene expression

A two-way interconnectedness exists between DNA supercoiling and gene transcription [176]. Negative supercoiling of the promoter region can stimulate transcription by facilitating the promoter melting and by increasing the DNA binding affinity of a protein regulator. Stochastic bursts of transcription appear to be a general characteristic of highly expressed genes, and supercoiling levels of the DNA template contributes to transcriptional bursting [177]. According to the twin supercoiling domain model, transcription-induced supercoiling can influence transcription of other nearby genes through a supercoiling relay. One such example is the activation of the *leu-500* promoter [176]. Supercoiling not only mediates gene-specific changes, but it also mediates large-scale changes in gene expression [178]. Global gene expression analysis in response to a loss of supercoiling identified 306 supercoiling-sensitive genes (SSGs) in *E*. *coli* [178]. SSGs are present throughout the chromosome and encode proteins with diverse functions. The topological organization of the nucleoid could allow independent expression of SSGs in different topological domains. A genome-scale map of unrestrained supercoiling showed that genomic regions have different steady-state supercoiling densities, indicating that the level of supercoiling differs in individual topological domains [170]. As a result, a change in supercoiling can result in domain-specific expression levels of SSGs, depending on the degree of supercoiling in each domain.

The effect of supercoiling on gene expression can be mediated by NAPs that directly or indirectly influence supercoiling. The effect of HU on gene expression appears to involve a change in supercoiling and perhaps a higher-order DNA organization [179]. A positive correlation between DNA gyrase binding and upregulation of the genes caused by the absence of HU suggests that changes in supercoiling are responsible for differential expression [47]. HU was also found to be responsible for a positional effect on gene expression by insulating transcriptional units by constraining transcription-induced supercoiling [180]. Point mutations in HUα dramatically changed the gene expression profile of *E*. *coli*, altering its morphology, physiology, and metabolism [171]. As a result, the mutant strain was more invasive of mammalian cells [181]. This dramatic effect was concomitant with nucleoid compaction and increased positive supercoiling. In contrast to the wild-type dimer, the mutant protein is an octamer that wraps DNA on its surface in a right-handed manner, restraining positive supercoils as opposed to wild-type HU [182]. These studies show that amino acid substitutions in HU can have a dramatic effect on nucleoid structure, and consequently, on gene expression, which in turn results in significant phenotypic changes.

Although HU appears to control gene expression by modulating supercoiling density, the exact molecular mechanism remains unknown. Since MukB and HU have emerged as critical players in long-range DNA interactions, it will be worthwhile to compare the effect of each of these two proteins on global gene expression. The impact of MukB on gene expression is yet to be analyzed.

### Current state and future perspective

Although studies of isolated nucleoids began in the 70s, a highly resolved and complete structure of the bacterial nucleoid is still not available. It remains a significant challenge even at 10 kb resolution, to accurately describe the hierarchical organization of chromosomal DNA resulting in the formation of a functional nucleoid. A single nucleotide resolution is even more difficult. However, we have significantly improved our understanding of the structural properties of the nucleoid. In summary, chromosomal DNA is a supercoiled molecule that folds into plectonemic loops. The supercoiling density is created and maintained by two topoisomerases (DNA gyrase and Topo I) with opposing functions. The NAPs organize DNA through DNA bending, bridging and looping activities. This organization together with transcriptional activities leads to higher-order organization of DNA into topologically and spatially distinct chromosomal interaction domains (microdomains) that are further organized as large size macrodomains. NAPs and macrodomain-specific proteins MukBEF and MatP appear to collaborate in the spatial organization. Supercoiling and topological domain formation also contribute to the proper regulation of gene transcription. The inherent bending of DNA due to Brownian motion and plectonemic supercoiling and DNA organization activities of NAPs and MukBEF provide condensation forces to sufficiently reduce the volume of the chromosomal DNA to fit within the cell volume. This process imparts a hierarchical organization, and the condensed chromosomal DNA results in a functional nucleoid with a helical ellipsoid shape.

Advances in imaging technologies can pave the way for direct visualization of higher-order nucleoid structure in vivo at high resolution. Recent electron microscopy methods that use high-pressure freezing and cryo-sectioning to preserve native ultrastructure [183], use improved DNA detection techniques for a sharp contrast [184], and allow 3D visualization with the cross-sectioning of cells using ion beams followed by 3D reconstruction [185] hold promises in driving new understanding of the 3D arrangement of the entire chromosomal DNA at high resolution. Although many issues regarding nucleoid fine structure and function remain to be resolved, some of the most interesting are the following:

- Is there a growth/environment dependent defined 3D structure of the DNA within the nucleoid?
- How are environmental cues transmitted to alter nucleoid structure?
- What is the nature of the supercoiling diffusion barriers that segregate the nucleoid into independent topological domains?
- What are the molecular mechanisms by which distant DNA-DNA contacts are made?
- What are the molecular mechanisms for the attachment of the membrane to nucleoid?

## Supporting information

S1 Text Version history of the text file. (XML)

S2 Text Peer reviews and response to reviews. (XML)

## References

[1] Stonington OG, Pettijohn DE. The folded genome of *Escherichia coli* isolated in a protein-DNA-RNA complex. Proc Natl Acad Sci U S A. 1971;68(1):6–9. Epub 1971/01/01. 10.1073/pnas.68.1.6 4924971 PMC391088

[2] Worcel A, Burgi E. On the structure of the folded chromosome of *Escherichia coli*. J Mol Biol. 1972;71(2):127–47. 10.1016/0022-2836(72)90342-7 . 4564477

[3] Dame RT, Tark-Dame M. Bacterial chromatin: converging views at different scales. Curr Opin Cell Biol. 2016;40:60–5. 10.1016/j.ceb.2016.02.015 . 26942688

[4] Kleckner N, Fisher JK, Stouf M, White MA, Bates D, Witz G. The bacterial nucleoid: nature, dynamics and sister segregation. Curr Opin Microbiol. 2014;22:127–37. Epub 2014/12/03. 10.1016/j.mib.2014.10.001 25460806 PMC4359759

[5] Bloomfield VA. DNA condensation by multivalent cations. Biopolymers. 1997;44(3):269–82. 10.1002/(SICI)1097-0282(1997)44:3<269::AID-BIP6>3.0.CO;2-T PubMed PMID: WOS:000073424300006. 9591479

[6] Trun N, Marko J. Architecture of a bacterial chromosome. Am Soc Microbiol News. 1998;64(5):276–83.

[7] Valens M, Penaud S, Rossignol M, Cornet F, Boccard F. Macrodomain organization of the *Escherichia coli* chromosome. EMBO J. 2004;23(21):4330–41. 10.1038/sj.emboj.7600434 15470498 PMC524398

[8] Fisher JK, Bourniquel A, Witz G, Weiner B, Prentiss M, Kleckner N. Four-dimensional imaging of E. coli nucleoid organization and dynamics in living cells. Cell. 2013;153(4):882–95. 10.1016/j.cell.2013.04.006 23623305 PMC3670778

[9] Le Gall A, Cattoni DI, Guilhas B, Mathieu-Demaziere C, Oudjedi L, Fiche JB, et al Bacterial partition complexes segregate within the volume of the nucleoid. Nat Commun. 2016;7:12107 10.1038/ncomms12107 27377966 PMC4935973

[10] Hadizadeh Yazdi N, Guet CC, Johnson RC, Marko JF. Variation of the folding and dynamics of the *Escherichia coli* chromosome with growth conditions. Mol Microbiol. 2012;86(6):1318–33. 10.1111/mmi.12071 23078205 PMC3524407

[11] Olins AL, Olins DE. Spheroid chromatin units (v bodies). Science. 1974;183(4122):330–2. Epub 1974/01/25. 10.1126/science.183.4122.330 . 4128918

[12] Luger K, Mader AW, Richmond RK, Sargent DF, Richmond TJ. Crystal structure of the nucleosome core particle at 2.8 A resolution. Nature. 1997;389(6648):251–60. Epub 1997/09/26. 10.1038/38444 . 9305837

[13] Khorasanizadeh S. The nucleosome: from genomic organization to genomic regulation. Cell. 2004;116(2):259–72. Epub 2004/01/28. 10.1016/s0092-8674(04)00044-3 . 14744436

[14] Talukder A, Ishihama A. Growth phase dependent changes in the structure and protein composition of nucleoid in *Escherichia coli*. Science China Life sciences. 2015;58(9):902–11. Epub 2015/07/26. 10.1007/s11427-015-4898-0 . 26208826

[15] Azam TA, Ishihama A. Twelve species of the nucleoid-associated protein from *Escherichia coli*. Sequence recognition specificity and DNA binding affinity. J Biol Chem. 1999;274(46):33105–13. Epub 1999/11/07. 10.1074/jbc.274.46.33105 . 10551881

[16] Ali Azam T, Iwata A, Nishimura A, Ueda S, Ishihama A. Growth phase-dependent variation in protein composition of the Escherichia coli nucleoid. J Bacteriol. 1999;181(20):6361–70. Epub 1999/10/09. 10515926 10.1128/jb.181.20.6361-6370.1999 PMC103771

[17] Swinger KK, Lemberg KM, Zhang Y, Rice PA. Flexible DNA bending in HU-DNA cocrystal structures. EMBO J. 2003;22(14):3749–60. 10.1093/emboj/cdg351 12853489 PMC165621

[18] Guo F, Adhya S. Spiral structure of *Escherichia coli* HUalphabeta provides foundation for DNA supercoiling. Proc Natl Acad Sci U S A. 2007;104(11):4309–14. Epub 2007/03/16. 10.1073/pnas.0611686104 17360520 PMC1838598

[19] Pinson V, Takahashi M, Rouviere-Yaniv J. Differential binding of the *Escherichia coli* HU, homodimeric forms and heterodimeric form to linear, gapped and cruciform DNA. J Mol Biol. 1999;287(3):485–97. Epub 1999/03/27. 10.1006/jmbi.1999.2631 . 10092454

[20] Lang B, Blot N, Bouffartigues E, Buckle M, Geertz M, Gualerzi CO, et al High-affinity DNA binding sites for H-NS provide a molecular basis for selective silencing within proteobacterial genomes. Nucleic Acids Research. 2007;35(18):6330–7. 10.1093/nar/gkm712 PubMed PMID: WOS:000250683600041. 17881364 PMC2094087

[21] Gulvady R, Gao Y, Kenney LJ, Yan J. A single molecule analysis of H-NS uncouples DNA binding affinity from DNA specificity. Nucleic Acids Res. 2018;46(19):10216–24. Epub 2018/09/22. 10.1093/nar/gky826 30239908 PMC6212787

[22] Craig NL, Nash HA. E. coli integration host factor binds to specific sites in DNA. Cell. 1984;39(3 Pt 2):707–16. 10.1016/0092-8674(84)90478-1 . 6096022

[23] Wang S, Cosstick R, Gardner JF, Gumport RI. The specific binding of *Escherichia coli* integration host factor involves both major and minor grooves of DNA. Biochemistry-Us. 1995;34(40):13082–90. Epub 1995/10/10. 10.1021/bi00040a020 . 7548068

[24] Shao Y, Feldman-Cohen LS, Osuna R. Functional characterization of the *Escherichia coli* Fis-DNA binding sequence. J Mol Biol. 2008;376(3):771–85. 10.1016/j.jmb.2007.11.101 18178221 PMC2292415

[25] Stella S, Cascio D, Johnson RC. The shape of the DNA minor groove directs binding by the DNA-bending protein Fis. Genes Dev. 2010;24(8):814–26. 10.1101/gad.1900610 20395367 PMC2854395

[26] Karas VO, Westerlaken I, Meyer AS. The DNA-Binding Protein from Starved Cells (Dps) Utilizes Dual Functions To Defend Cells against Multiple Stresses. J Bacteriol. 2015;197(19):3206–15. Epub 2015/07/29. 10.1128/JB.00475-15 26216848 PMC4560292

[27] Mercier R, Petit MA, Schbath S, Robin S, El Karoui M, Boccard F, et al The MatP/matS Site-Specific System Organizes the Terminus Region of the E-coli Chromosome into a Macrodomain. Cell. 2008;135(3):475–85. 10.1016/j.cell.2008.08.031 PubMed PMID: WOS:000260536300018. 18984159

[28] Rouviere-Yaniv J, Gros F. Characterization of a novel, low-molecular-weight DNA-binding protein from *Escherichia coli*. Proc Natl Acad Sci U S A. 1975;72(9):3428–32. Epub 1975/09/01. 10.1073/pnas.72.9.3428 1103148 PMC433007

[29] Suryanarayana T, Subramanian AR. Specific association of two homologous DNA-binding proteins to the native 30-S ribosomal subunits of *Escherichia coli*. Biochimica et biophysica acta. 1978;520(2):342–57. Epub 1978/09/27. 10.1016/0005-2787(78)90232-0 . 213117

[30] Mende L, Timm B, Subramanian R. Primary structures of two homologous ribosome-associated DNA-binding proteins of *Escherichia coli*. FEBS Lett. 1978;96(2):395–8. 10.1016/0014-5793(78)80446-3 . 215461

[31] Megraw TL, Chae CB. Functional complementarity between the HMG1-like yeast mitochondrial histone HM and the bacterial histone-like protein HU. J Biol Chem. 1993;268(17):12758–63. Epub 1993/06/15. . 8509411

[32] Paull TT, Johnson RC. DNA looping by Saccharomyces cerevisiae high mobility group proteins NHP6A/B. Consequences for nucleoprotein complex assembly and chromatin condensation. J Biol Chem. 1995;270(15):8744–54. Epub 1995/04/14. 10.1074/jbc.270.15.8744 . 7721780

[33] Kamashev D, Rouviere-Yaniv J. The histone-like protein HU binds specifically to DNA recombination and repair intermediates. EMBO J. 2000;19(23):6527–35. Epub 2000/12/02. 10.1093/emboj/19.23.6527 11101525 PMC305869

[34] Shindo H, Furubayashi A, Shimizu M, Miyake M, Imamoto F. Preferential binding of E.coli histone-like protein HU alpha to negatively supercoiled DNA. Nucleic Acids Res. 1992;20(7):1553–8. 10.1093/nar/20.7.1553 1579448 PMC312237

[35] Pontiggia A, Negri A, Beltrame M, Bianchi ME. Protein HU binds specifically to kinked DNA. Mol Microbiol. 1993;7(3):343–50. 10.1111/j.1365-2958.1993.tb01126.x . 8459763

[36] Bonnefoy E, Takahashi M, Yaniv JR. DNA-binding parameters of the HU protein of *Escherichia coli* to cruciform DNA. J Mol Biol. 1994;242(2):116–29. 10.1006/jmbi.1994.1563 . 8089835

[37] Castaing B, Zelwer C, Laval J, Boiteux S. HU protein of *Escherichia coli* binds specifically to DNA that contains single-strand breaks or gaps. J Biol Chem. 1995;270(17):10291–6. 10.1074/jbc.270.17.10291 . 7730334

[38] Lyubchenko YL, Shlyakhtenko LS, Aki T, Adhya S. Atomic force microscopic demonstration of DNA looping by GalR and HU. Nucleic Acids Res. 1997;25(4):873–6. 10.1093/nar/25.4.873 9016640 PMC146491

[39] Swinger KK, Rice PA. Structure-based analysis of HU-DNA binding. J Mol Biol. 2007;365(4):1005–16. Epub 2006/11/14. 10.1016/j.jmb.2006.10.024 17097674 PMC1945228

[40] Hammel M, Amlanjyoti D, Reyes FE, Chen JH, Parpana R, Tang HY, et al HU multimerization shift controls nucleoid compaction. Sci Adv. 2016;2(7):e1600650 10.1126/sciadv.1600650 27482541 PMC4966879

[41] Kano Y, Goshima N, Wada M, Imamoto F. Participation of hup gene product in replicative transposition of Mu phage in *Escherichia coli*. Gene. 1989;76(2):353–8. 10.1016/0378-1119(89)90175-3 . 2666261

[42] Ogura T, Niki H, Kano Y, Imamoto F, Hiraga S. Maintenance of plasmids in HU and IHF mutants of *Escherichia coli*. Mol Gen Genet. 1990;220(2):197–203. 10.1007/bf00260482 . 2183003

[43] Hwang DS, Kornberg A. Opening of the replication origin of *Escherichia coli* by DnaA protein with protein HU or IHF. J Biol Chem. 1992;267(32):23083–6. Epub 1992/11/15. . 1429655

[44] Macvanin M, Edgar R, Cui F, Trostel A, Zhurkin V, Adhya S. Noncoding RNAs binding to the nucleoid protein HU in *Escherichia coli*. J Bacteriol. 2012;194(22):6046–55. Epub 2012/09/04. 10.1128/JB.00961-12 22942248 PMC3486375

[45] van Noort J, Verbrugge S, Goosen N, Dekker C, Dame RT. Dual architectural roles of HU: formation of flexible hinges and rigid filaments. Proc Natl Acad Sci U S A. 2004;101(18):6969–74. Epub 2004/05/01. 10.1073/pnas.0308230101 15118104 PMC406450

[46] Kahramanoglou C, Seshasayee AS, Prieto AI, Ibberson D, Schmidt S, Zimmermann J, et al Direct and indirect effects of H-NS and Fis on global gene expression control in *Escherichia coli*. Nucleic Acids Res. 2011;39(6):2073–91. 10.1093/nar/gkq934 21097887 PMC3064808

[47] Prieto AI, Kahramanoglou C, Ali RM, Fraser GM, Seshasayee AS, Luscombe NM. Genomic analysis of DNA binding and gene regulation by homologous nucleoid-associated proteins IHF and HU in *Escherichia coli* K12. Nucleic Acids Res. 2012;40(8):3524–37. Epub 2011/12/20. 10.1093/nar/gkr1236 22180530 PMC3333857

[48] Sarkar R, Rybenkov VV. A Guide to Magnetic Tweezers and Their Applications. Front Phys. 2016;4. doi: ARTN 48. PubMed PMID: WOS:000389259500002. 10.3389/fphy.2016.00048

[49] Rice PA, Yang S, Mizuuchi K, Nash HA. Crystal structure of an IHF-DNA complex: a protein-induced DNA U-turn. Cell. 1996;87(7):1295–306. 10.1016/s0092-8674(00)81824-3 . 8980235

[50] Murtin C, Engelhorn M, Geiselmann J, Boccard F. A quantitative UV laser footprinting analysis of the interaction of IHF with specific binding sites: re-evaluation of the effective concentration of IHF in the cell. J Mol Biol. 1998;284(4):949–61. Epub 1998/12/05. 10.1006/jmbi.1998.2256 . 9837718

[51] Ditto MD, Roberts D, Weisberg RA. Growth phase variation of integration host factor level in *Escherichia coli*. J Bacteriol. 1994;176(12):3738–48. Epub 1994/06/01. 10.1128/jb.176.12.3738-3748.1994 8206852 PMC205563

[52] Lin J, Chen H, Droge P, Yan J. Physical organization of DNA by multiple non-specific DNA-binding modes of integration host factor (IHF). PLoS One. 2012;7(11):e49885 10.1371/journal.pone.0049885 23166787 PMC3498176

[53] Jacquet M, Cukier-Kahn R, Pla J, Gros F. A thermostable protein factor acting on in vitro DNA transcription. Biochem Biophys Res Commun. 1971;45(6):1597–607. 10.1016/0006-291x(71)90204-x . 4942735

[54] Cukier-Kahn R, Jacquet M, Gros F. Two heat-resistant, low molecular weight proteins from *Escherichia coli* that stimulate DNA-directed RNA synthesis. Proc Natl Acad Sci U S A. 1972;69(12):3643–7. 10.1073/pnas.69.12.3643 4566454 PMC389839

[55] Spassky A, Buc HC. Physico-chemical properties of a DNA binding protein: *Escherichia coli* factor H1. Eur J Biochem. 1977;81(1):79–90. 10.1111/j.1432-1033.1977.tb11929.x . 338303

[56] Varshavsky AJ, Nedospasov SA, Bakayev VV, Bakayeva TG, Georgiev GP. Histone-like proteins in the purified *Escherichia coli* deoxyribonucleoprotein. Nucleic Acids Res. 1977;4(8):2725–45. 10.1093/nar/4.8.2725 333393 PMC342604

[57] Falconi M, Gualtieri MT, La Teana A, Losso MA, Pon CL. Proteins from the prokaryotic nucleoid: primary and quaternary structure of the 15-kD *Escherichia coli* DNA binding protein H-NS. Mol Microbiol. 1988;2(3):323–9. 10.1111/j.1365-2958.1988.tb00035.x . 3135462

[58] Ueguchi C, Suzuki T, Yoshida T, Tanaka K, Mizuno T. Systematic mutational analysis revealing the functional domain organization of *Escherichia coli* nucleoid protein H-NS. J Mol Biol. 1996;263(2):149–62. 10.1006/jmbi.1996.0566 . 8913298

[59] Rimsky S, Zuber F, Buckle M, Buc H. A molecular mechanism for the repression of transcription by the H-NS protein. Mol Microbiol. 2001;42(5):1311–23. 10.1046/j.1365-2958.2001.02706.x . 11886561

[60] Bouffartigues E, Buckle M, Badaut C, Travers A, Rimsky S. H-NS cooperative binding to high-affinity sites in a regulatory element results in transcriptional silencing. Nat Struct Mol Biol. 2007;14(5):441–8. Epub 2007/04/17. 10.1038/nsmb1233 . 17435766

[61] Amit R, Oppenheim AB, Stavans J. Increased bending rigidity of single DNA molecules by H-NS, a temperature and osmolarity sensor. Biophys J. 2003;84(4):2467–73. 10.1016/S0006-3495(03)75051-6 12668454 PMC1302812

[62] Dame RT, Noom MC, Wuite GJ. Bacterial chromatin organization by H-NS protein unravelled using dual DNA manipulation. Nature. 2006;444(7117):387–90. 10.1038/nature05283 . 17108966

[63] Dame RT, Wyman C, Goosen N. H-NS mediated compaction of DNA visualised by atomic force microscopy. Nucleic Acids Res. 2000;28(18):3504–10. 10.1093/nar/28.18.3504 10982869 PMC110753

[64] Liu Y, Chen H, Kenney LJ, Yan J. A divalent switch drives H-NS/DNA-binding conformations between stiffening and bridging modes. Genes Dev. 2010;24(4):339–44. 10.1101/gad.1883510 20159954 PMC2816733

[65] van der Valk RA, Vreede J, Qin L, Moolenaar GF, Hofmann A, Goosen N, et al Mechanism of environmentally driven conformational changes that modulate H-NS DNA-bridging activity. Elife. 2017;6. doi: e27369 10.7554/eLife.27369 PubMed PMID: WOS:000413181000001. PMC5647153 28949292

[66] Yamada H, Muramatsu S, Mizuno T. An Escherichia-Coli Protein That Preferentially Binds to Sharply Curved DNA. J Biochem-Tokyo. 1990;108(3):420–5. PubMed PMID: WOS:A1990DX38200014. 10.1093/oxfordjournals.jbchem.a123216 2126011

[67] Martin-Orozco N, Touret N, Zaharik ML, Park E, Kopelman R, Miller S, et al Visualization of vacuolar acidification-induced transcription of genes of pathogens inside macrophages. Molecular biology of the cell. 2006;17(1):498–510. Epub 2005/10/28. 10.1091/mbc.E04-12-1096 16251362 PMC1345685

[68] Winardhi RS, Yan J, Kenney LJ. H-NS Regulates Gene Expression and Compacts the Nucleoid: Insights from Single-Molecule Experiments. Biophys J. 2015;109(7):1321–9. 10.1016/j.bpj.2015.08.016 26445432 PMC4601063

[69] Walthers D, Li Y, Liu Y, Anand G, Yan J, Kenney LJ. Salmonella enterica response regulator SsrB relieves H-NS silencing by displacing H-NS bound in polymerization mode and directly activates transcription. J Biol Chem. 2011;286(3):1895–902. Epub 2010/11/10. 10.1074/jbc.M110.164962 21059643 PMC3023485

[70] Gao Y, Foo YH, Winardhi RS, Tang Q, Yan J, Kenney LJ. Charged residues in the H-NS linker drive DNA binding and gene silencing in single cells. Proc Natl Acad Sci U S A. 2017;114(47):12560–5. Epub 2017/11/08. 10.1073/pnas.1716721114 29109287 PMC5703333

[71] Hancock SP, Stella S, Cascio D, Johnson RC. DNA Sequence Determinants Controlling Affinity, Stability and Shape of DNA Complexes Bound by the Nucleoid Protein Fis. PLoS One. 2016;11(3):e0150189 10.1371/journal.pone.0150189 26959646 PMC4784862

[72] Kostrewa D, Granzin J, Koch C, Choe HW, Raghunathan S, Wolf W, et al Three-dimensional structure of the E. coli DNA-binding protein FIS. Nature. 1991;349(6305):178–80. 10.1038/349178a0 . 1986310

[73] Kostrewa D, Granzin J, Stock D, Choe HW, Labahn J, Saenger W. Crystal structure of the factor for inversion stimulation FIS at 2.0 A resolution. J Mol Biol. 1992;226(1):209–26. 10.1016/0022-2836(92)90134-6 . 1619650

[74] Cho BK, Knight EM, Barrett CL, Palsson BO. Genome-wide analysis of Fis binding in *Escherichia coli* indicates a causative role for A-/AT-tracts. Genome Res. 2008;18(6):900–10. 10.1101/gr.070276.107 18340041 PMC2413157

[75] Travers A, Muskhelishvili G. DNA microloops and microdomains: a general mechanism for transcription activation by torsional transmission. J Mol Biol. 1998;279(5):1027–43. 10.1006/jmbi.1998.1834 . 9642081

[76] Skoko D, Yan J, Johnson RC, Marko JF. Low-force DNA condensation and discontinuous high-force decondensation reveal a loop-stabilizing function of the protein Fis. Physical review letters. 2005;95(20):208101 10.1103/PhysRevLett.95.208101 . 16384101

[77] Skoko D, Yoo D, Bai H, Schnurr B, Yan J, McLeod SM, et al Mechanism of chromosome compaction and looping by the *Escherichia coli* nucleoid protein Fis. J Mol Biol. 2006;364(4):777–98. 10.1016/j.jmb.2006.09.043 17045294 PMC1988847

[78] Johnson RC, Simon MI. Hin-mediated site-specific recombination requires two 26 bp recombination sites and a 60 bp recombinational enhancer. Cell. 1985;41(3):781–91. 10.1016/s0092-8674(85)80059-3 . 2988787

[79] Kahmann R, Rudt F, Koch C, Mertens G. G-Inversion in Bacteriophage-Mu-DNA Is Stimulated by a Site within the Invertase Gene and a Host Factor. Cell. 1985;41(3):771–80. 10.1016/s0092-8674(85)80058-1 PubMed PMID: WOS:A1985AMS1500016. 3159478

[80] Pettijohn DE, Hecht R. RNA molecules bound to the folded bacterial genome stabilize DNA folds and segregate domains of supercoiling. Cold Spring Harbor symposia on quantitative biology. 1974;38:31–41. Epub 1974/01/01. 10.1101/sqb.1974.038.01.006 . 4598638

[81] Ohniwa RL, Morikawa K, Takeshita SL, Kim J, Ohta T, Wada C, et al Transcription-coupled nucleoid architecture in bacteria. Genes Cells. 2007;12(10):1141–52. 10.1111/j.1365-2443.2007.01125.x . 17903174

[82] Balandina A, Kamashev D, Rouviere-Yaniv J. The bacterial histone-like protein HU specifically recognizes similar structures in all nucleic acids. DNA, RNA, and their hybrids. J Biol Chem. 2002;277(31):27622–8. Epub 2002/05/15. 10.1074/jbc.M201978200 . 12006568

[83] Balandina A, Claret L, Hengge-Aronis R, Rouviere-Yaniv J. The *Escherichia coli* histone-like protein HU regulates rpoS translation. Mol Microbiol. 2001;39(4):1069–79. Epub 2001/03/17. 10.1046/j.1365-2958.2001.02305.x . 11251825

[84] Qian Z, Macvanin M, Dimitriadis EK, He X, Zhurkin V, Adhya S. A New Noncoding RNA Arranges Bacterial Chromosome Organization. MBio. 2015;6(4). Epub 2015/08/27. 10.1128/mBio.00998-15 26307168 PMC4550694

[85] Qian Z, Zhurkin VB, Adhya S. DNA-RNA interactions are critical for chromosome condensation in *Escherichia coli*. Proc Natl Acad Sci U S A. 2017;114(46):12225–30. Epub 2017/11/01. 10.1073/pnas.1711285114 29087325 PMC5699063

[86] Bauer WR, Crick FH, White JH. Supercoiled DNA. Sci Am. 1980;243(1):100–13. Epub 1980/07/01. . 6256851

[87] Sinden R. DNA Structure and Function. San Diego: Academic Press; 1994.

[88] Bates AD, Maxwell A. DNA Topology. Oxford: Oxford University Press; 2005.

[89] Sinden RR, Carlson JO, Pettijohn DE. Torsional tension in the DNA double helix measured with trimethylpsoralen in living E. coli cells: analogous measurements in insect and human cells. Cell. 1980;21(3):773–83. 10.1016/0092-8674(80)90440-7 . 6254668

[90] Griffith JD. Visualization of prokaryotic DNA in a regularly condensed chromatin-like fiber. Proc Natl Acad Sci U S A. 1976;73(2):563–7. 10.1073/pnas.73.2.563 1108025 PMC335950

[91] Postow L, Hardy CD, Arsuaga J, Cozzarelli NR. Topological domain structure of the *Escherichia coli* chromosome. Genes Dev. 2004;18(14):1766–79. 10.1101/gad.1207504 15256503 PMC478196

[92] Bliska JB, Cozzarelli NR. Use of site-specific recombination as a probe of DNA structure and metabolism in vivo. J Mol Biol. 1987;194(2):205–18. 10.1016/0022-2836(87)90369-x . 3039150

[93] Holmes VF, Cozzarelli NR. Closing the ring: Links between SMC proteins and chromosome partitioning, condensation, and supercoiling. P Natl Acad Sci USA. 2000;97(4):1322–4. 10.1073/pnas.040576797 PubMed PMID: WOS:000085409600003. 10677457 PMC34294

[94] Champoux JJ. DNA topoisomerases: structure, function, and mechanism. Annu Rev Biochem. 2001;70:369–413. 10.1146/annurev.biochem.70.1.369 . 11395412

[95] Gellert M, Mizuuchi K, Odea MH, Nash HA. DNA Gyrase—Enzyme That Introduces Superhelical Turns into DNA. P Natl Acad Sci USA. 1976;73(11):3872–6. 10.1073/pnas.73.11.3872 PubMed PMID: WOS:A1976CN11500019. 186775 PMC431247

[96] Depew RE, Liu LF, Wang JC. Interaction between DNA and Escherichia-Coli Protein-Omega—Formation of a Complex between Single-Stranded-DNA and Omega-Protein. Journal of Biological Chemistry. 1978;253(2):511–8. PubMed PMID: WOS:A1978EK02600037. 338610

[97] Kirkegaard K, Wang JC. *Escherichia coli* DNA topoisomerase I catalyzed linking of single-stranded rings of complementary base sequences. Nucleic Acids Res. 1978;5(10):3811–20. Epub 1978/10/01. 10.1093/nar/5.10.3811 214763 PMC342711

[98] Raji A, Zabel DJ, Laufer CS, Depew RE. Genetic-Analysis of Mutations That Compensate for Loss of Escherichia-Coli DNA Topoisomerase-I. Journal of Bacteriology. 1985;162(3):1173–9. PubMed PMID: WOS:A1985AJL5700047. 2987184 10.1128/jb.162.3.1173-1179.1985 PMC215900

[99] Dean F, Krasnow MA, Otter R, Matzuk MM, Spengler SJ, Cozzarelli NR. *Escherichia coli* type-1 topoisomerases: identification, mechanism, and role in recombination. Cold Spring Harbor symposia on quantitative biology. 1983;47 Pt 2:769–77. 10.1101/sqb.1983.047.01.088 . 6305585

[100] Zechiedrich EL, Khodursky AB, Bachellier S, Schneider R, Chen D, Lilley DM, et al Roles of topoisomerases in maintaining steady-state DNA supercoiling in *Escherichia coli*. J Biol Chem. 2000;275(11):8103–13. 10.1074/jbc.275.11.8103 . 10713132

[101] Kato J, Nishimura Y, Imamura R, Niki H, Hiraga S, Suzuki H. New topoisomerase essential for chromosome segregation in E. coli. Cell. 1990;63(2):393–404. 10.1016/0092-8674(90)90172-b . 2170028

[102] Liu LF, Wang JC. Supercoiling of the DNA template during transcription. Proc Natl Acad Sci U S A. 1987;84(20):7024–7. 10.1073/pnas.84.20.7024 2823250 PMC299221

[103] Kouzine F, Liu J, Sanford S, Chung HJ, Levens D. The dynamic response of upstream DNA to transcription-generated torsional stress. Nat Struct Mol Biol. 2004;11(11):1092–100. Epub 2004/10/27. 10.1038/nsmb848 . 15502847

[104] Rouviere-Yaniv J, Yaniv M, Germond JE. E. coli DNA binding protein HU forms nucleosomelike structure with circular double-stranded DNA. Cell. 1979;17(2):265–74. Epub 1979/06/01. 10.1016/0092-8674(79)90152-1 . 222478

[105] Broyles SS, Pettijohn DE. Interaction of the *Escherichia coli* HU protein with DNA. Evidence for formation of nucleosome-like structures with altered DNA helical pitch. J Mol Biol. 1986;187(1):47–60. Epub 1986/01/05. 10.1016/0022-2836(86)90405-5 . 3514923

[106] Kundukad B, Cong P, van der Maarel JR, Doyle PS. Time-dependent bending rigidity and helical twist of DNA by rearrangement of bound HU protein. Nucleic Acids Res. 2013;41(17):8280–8. 10.1093/nar/gkt593 23828037 PMC3783175

[107] Tupper AE, Owen-Hughes TA, Ussery DW, Santos DS, Ferguson DJ, Sidebotham JM, et al The chromatin-associated protein H-NS alters DNA topology in vitro. EMBO J. 1994;13(1):258–68. 8306968 10.1002/j.1460-2075.1994.tb06256.x PMC394800

[108] Schneider R, Lurz R, Luder G, Tolksdorf C, Travers A, Muskhelishvili G. An architectural role of the *Escherichia coli* chromatin protein FIS in organising DNA. Nucleic Acids Res. 2001;29(24):5107–14. 10.1093/nar/29.24.5107 11812843 PMC97572

[109] Schneider R, Travers A, Kutateladze T, Muskhelishvili G. A DNA architectural protein couples cellular physiology and DNA topology in *Escherichia coli*. Mol Microbiol. 1999;34(5):953–64. 10.1046/j.1365-2958.1999.01656.x . 10594821

[110] Bensaid A, Almeida A, Drlica K, Rouviere-Yaniv J. Cross-talk between topoisomerase I and HU in *Escherichia coli*. J Mol Biol. 1996;256(2):292–300. Epub 1996/02/23. 10.1006/jmbi.1996.0086 . 8594197

[111] Malik M, Bensaid A, Rouviere-Yaniv J, Drlica K. Histone-like protein HU and bacterial DNA topology: suppression of an HU deficiency by gyrase mutations. J Mol Biol. 1996;256(1):66–76. Epub 1996/02/16. 10.1006/jmbi.1996.0068 . 8609614

[112] Marians KJ. DNA gyrase-catalyzed decatenation of multiply linked DNA dimers. J Biol Chem. 1987;262(21):10362–8. Epub 1987/07/25. . 3038875

[113] Sinden RR, Pettijohn DE. Chromosomes in living *Escherichia coli* cells are segregated into domains of supercoiling. Proc Natl Acad Sci U S A. 1981;78(1):224–8. Epub 1981/01/01. 10.1073/pnas.78.1.224 6165987 PMC319024

[114] Higgins NP, Yang XL, Fu QQ, Roth JR. Surveying a supercoil domain by using the gamma delta resolution system in Salmonella typhimurium. Journal of Bacteriology. 1996;178(10):2825–35. 10.1128/jb.178.10.2825-2835.1996 PubMed PMID: WOS:A1996UL27500013. 8631670 PMC178017

[115] Yan Y, Ding Y, Leng F, Dunlap D, Finzi L. Protein-mediated loops in supercoiled DNA create large topological domains. Nucleic Acids Res. 2018;46(9):4417–24. Epub 2018/03/15. 10.1093/nar/gky153 29538766 PMC5961096

[116] Leng F, Chen B, Dunlap DD. Dividing a supercoiled DNA molecule into two independent topological domains. Proc Natl Acad Sci U S A. 2011;108(50):19973–8. 10.1073/pnas.1109854108 22123985 PMC3250177

[117] Moulin L, Rahmouni AR, Boccard F. Topological insulators inhibit diffusion of transcription-induced positive supercoils in the chromosome of *Escherichia coli*. Mol Microbiol. 2005;55(2):601–10. Epub 2005/01/22. 10.1111/j.1365-2958.2004.04411.x . 15659173

[118] Dimri GP, Rudd KE, Morgan MK, Bayat H, Ames GFL. Physical Mapping of Repetitive Extragenic Palindromic Sequences in Escherichia-Coli and Phylogenetic Distribution among Escherichia-Coli Strains and Other Enteric Bacteria. Journal of Bacteriology. 1992;174(14):4583–93. 10.1128/jb.174.14.4583-4593.1992 :A1992JE39900007. 1624447 PMC206253

[119] Booker BM, Deng S, Higgins NP. DNA topology of highly transcribed operons in Salmonella enterica serovar Typhimurium. Mol Microbiol. 2010;78(6):1348–64. Epub 2010/12/15. 10.1111/j.1365-2958.2010.07394.x . 21143310

[120] Deng S, Stein RA, Higgins NP. Organization of supercoil domains and their reorganization by transcription. Molecular Microbiology. 2005;57(6):1511–21. 10.1111/j.1365-2958.2005.04796.x PubMed PMID: WOS:000231610600001. 16135220 PMC1382059

[121] Deng S, Stein RA, Higgins NP. Transcription-induced barriers to supercoil diffusion in the Salmonella typhimurium chromosome. Proc Natl Acad Sci U S A. 2004;101(10):3398–403. Epub 2004/03/03. 10.1073/pnas.0307550101 14993611 PMC373473

[122] Dekker J, Rippe K, Dekker M, Kleckner N. Capturing chromosome conformation. Science. 2002;295(5558):1306–11. 10.1126/science.1067799 PubMed PMID: WOS:000173926000047. 11847345

[123] Lieberman-Aiden E, van Berkum NL, Williams L, Imakaev M, Ragoczy T, Telling A, et al Comprehensive mapping of long-range interactions reveals folding principles of the human genome. Science. 2009;326(5950):289–93. Epub 2009/10/10. 10.1126/science.1181369 19815776 PMC2858594

[124] Lioy VS, Cournac A, Marbouty M, Duigou S, Mozziconacci J, Espeli O, et al Multiscale Structuring of the E. coli Chromosome by Nucleoid-Associated and Condensin Proteins. Cell. 2018;172(4):771–83 e18. Epub 2018/01/24. 10.1016/j.cell.2017.12.027 . 29358050

[125] Le TB, Imakaev MV, Mirny LA, Laub MT. High-resolution mapping of the spatial organization of a bacterial chromosome. Science. 2013;342(6159):731–4. Epub 2013/10/26. 10.1126/science.1242059 24158908 PMC3927313

[126] Wang X, Le TB, Lajoie BR, Dekker J, Laub MT, Rudner DZ. Condensin promotes the juxtaposition of DNA flanking its loading site in Bacillus subtilis. Genes Dev. 2015;29(15):1661–75. Epub 2015/08/09. 10.1101/gad.265876.115 26253537 PMC4536313

[127] Dekker J, Heard E. Structural and functional diversity of Topologically Associating Domains. FEBS Lett. 2015;589(20 Pt A):2877–84. Epub 2015/09/09. 10.1016/j.febslet.2015.08.044 26348399 PMC4598308

[128] Niki H, Hiraga S. Polar localization of the replication origin and terminus in *Escherichia coli* nucleoids during chromosome partitioning. Genes Dev. 1998;12(7):1036–45. Epub 1998/05/09. 10.1101/gad.12.7.1036 9531540 PMC316681

[129] Niki H, Yamaichi Y, Hiraga S. Dynamic organization of chromosomal DNA in *Escherichia coli*. Genes Dev. 2000;14(2):212–23. Epub 2000/02/01. 10652275 PMC316355

[130] Boccard F, Esnault E, Valens M. Spatial arrangement and macrodomain organization of bacterial chromosomes. Mol Microbiol. 2005;57(1):9–16. Epub 2005/06/14. 10.1111/j.1365-2958.2005.04651.x . 15948945

[131] Duigou S, Boccard F. Long range chromosome organization in *Escherichia coli*: The position of the replication origin defines the non-structured regions and the Right and Left macrodomains. PLoS Genet. 2017;13(5):e1006758 10.1371/journal.pgen.1006758 28486476 PMC5441646

[132] Nolivos S, Upton AL, Badrinarayanan A, Muller J, Zawadzka K, Wiktor J, et al MatP regulates the coordinated action of topoisomerase IV and MukBEF in chromosome segregation. Nat Commun. 2016;7:10466 Epub 2016/01/29. 10.1038/ncomms10466 26818444 PMC4738335

[133] Dupaigne P, Tonthat NK, Espeli O, Whitfill T, Boccard F, Schumacher MA. Molecular Basis for a Protein-Mediated DNA-Bridging Mechanism that Functions in Condensation of the E. coli Chromosome. Molecular Cell. 2012;48(4):560–71. 10.1016/j.molcel.2012.09.009 PubMed PMID: WOS:000311919500009. 23084832 PMC7505563

[134] Woo JS, Lim JH, Shin HC, Suh MK, Ku B, Lee KH, et al Structural studies of a bacterial condensin complex reveal ATP-dependent disruption of intersubunit interactions. Cell. 2009;136(1):85–96. 10.1016/j.cell.2008.10.050 . 19135891

[135] Fennell-Fezzie R, Gradia SD, Akey D, Berger JM. The MukF subunit of *Escherichia coli* condensin: architecture and functional relationship to kleisins. EMBO J. 2005;24(11):1921–30. 10.1038/sj.emboj.7600680 15902272 PMC1142612

[136] Petrushenko ZM, Lai CH, Rybenkov VV. Antagonistic interactions of kleisins and DNA with bacterial Condensin MukB. J Biol Chem. 2006;281(45):34208–17. 10.1074/jbc.M606723200 16982609 PMC1634889

[137] Badrinarayanan A, Reyes-Lamothe R, Uphoff S, Leake MC, Sherratt DJ. In vivo architecture and action of bacterial structural maintenance of chromosome proteins. Science. 2012;338(6106):528–31. 10.1126/science.1227126 23112333 PMC3807729

[138] Kumar R, Grosbart M, Nurse P, Bahng S, Wyman CL, Marians KJ. The bacterial condensin MukB compacts DNA by sequestering supercoils and stabilizing topologically isolated loops. J Biol Chem. 2017;292(41):16904–20. 10.1074/jbc.M117.803312 28842486 PMC5641887

[139] Uhlmann F. SMC complexes: from DNA to chromosomes. Nat Rev Mol Cell Biol. 2016;17(7):399–412. 10.1038/nrm.2016.30 . 27075410

[140] Melby TE, Ciampaglio CN, Briscoe G, Erickson HP. The symmetrical structure of structural maintenance of chromosomes (SMC) and MukB proteins: Long, antiparallel coiled coils, folded at a flexible hinge. Journal of Cell Biology. 1998;142(6):1595–604. 10.1083/jcb.142.6.1595 PubMed PMID: WOS:000076116400016. 9744887 PMC2141774

[141] Niki H, Imamura R, Kitaoka M, Yamanaka K, Ogura T, Hiraga S. E.coli MukB protein involved in chromosome partition forms a homodimer with a rod-and-hinge structure having DNA binding and ATP/GTP binding activities. EMBO J. 1992;11(13):5101–9. 1464330 10.1002/j.1460-2075.1992.tb05617.x PMC556988

[142] Yamazoe M, Onogi T, Sunako Y, Niki H, Yamanaka K, Ichimura T, et al Complex formation of MukB, MukE and MukF proteins involved in chromosome partitioning in *Escherichia coli*. EMBO J. 1999;18(21):5873–84. Epub 1999/11/02. 10.1093/emboj/18.21.5873 10545099 PMC1171653

[143] Li YY, Stewart NK, Berger AJ, Vos S, Schoeffler AJ, Berger JM, et al *Escherichia coli* condensin MukB stimulates topoisomerase IV activity by a direct physical interaction. P Natl Acad Sci USA. 2010;107(44):18832–7. 10.1073/pnas.1008678107 PubMed PMID: WOS:000283749000021. 20921377 PMC2973889

[144] Nicolas E, Upton AL, Uphoff S, Henry O, Badrinarayanan A, Sherratt D. The SMC complex MukBEF recruits topoisomerase IV to the origin of replication region in live *Escherichia coli*. MBio. 2014;5(1):e01001–13. 10.1128/mBio.01001-13 24520061 PMC3950513

[145] Vos SM, Stewart NK, Oakley MG, Berger JM. Structural basis for the MukB-topoisomerase IV interaction and its functional implications in vivo. EMBO J. 2013;32(22):2950–62. 10.1038/emboj.2013.218 24097060 PMC3832749

[146] Zawadzki P, Stracy M, Ginda K, Zawadzka K, Lesterlin C, Kapanidis AN, et al The Localization and Action of Topoisomerase IV in *Escherichia coli* Chromosome Segregation Is Coordinated by the SMC Complex, MukBEF. Cell reports. 2015;13(11):2587–96. Epub 2015/12/22. 10.1016/j.celrep.2015.11.034 26686641 PMC5061553

[147] Badrinarayanan A, Lesterlin C, Reyes-Lamothe R, Sherratt D. The *Escherichia coli* SMC complex, MukBEF, shapes nucleoid organization independently of DNA replication. J Bacteriol. 2012;194(17):4669–76. 10.1128/JB.00957-12 22753058 PMC3415497

[148] Baxter J, Oliver AW, Schalbetter SA. Are SMC Complexes Loop Extruding Factors? Linking Theory With Fact. Bioessays. 2019;41(1):e1800182 Epub 2018/12/07. 10.1002/bies.201800182 . 30506702

[149] Zawadzka K, Zawadzki P, Baker R, Rajasekar KV, Wagner F, Sherratt DJ, et al MukB ATPases are regulated independently by the N- and C-terminal domains of MukF kleisin. Elife. 2018;7 Epub 2018/01/13. 10.7554/eLife.31522 29323635 PMC5812716

[150] Sawitzke JA, Austin S. Suppression of chromosome segregation defects of *Escherichia coli* muk mutants by mutations in topoisomerase I. Proc Natl Acad Sci U S A. 2000;97(4):1671–6. 10.1073/pnas.030528397 10660686 PMC26494

[151] Adachi S, Hiraga S. Mutants suppressing novobiocin hypersensitivity of a mukB null mutation. J Bacteriol. 2003;185(13):3690–5. 10.1128/JB.185.13.3690-3695.2003 12813060 PMC161581

[152] Petrushenko ZM, Lai CH, Rai R, Rybenkov VV. DNA reshaping by MukB. Right-handed knotting, left-handed supercoiling. J Biol Chem. 2006;281(8):4606–15. 10.1074/jbc.M504754200 16368697 PMC1633270

[153] Gaal T, Bratton BP, Sanchez-Vazquez P, Sliwicki A, Sliwicki K, Vegel A, et al Colocalization of distant chromosomal loci in space in E. coli: a bacterial nucleolus. Genes Dev. 2016;30(20):2272–85. Epub 2016/11/30. 10.1101/gad.290312.116 27898392 PMC5110994

[154] Jin DJ, Cabrera JE. Coupling the distribution of RNA polymerase to global gene regulation and the dynamic structure of the bacterial nucleoid in *Escherichia coli*. J Struct Biol. 2006;156(2):284–91. Epub 2006/08/29. 10.1016/j.jsb.2006.07.005 . 16934488

[155] Qian Z, Dimitriadis EK, Edgar R, Eswaramoorthy P, Adhya S. Galactose repressor mediated intersegmental chromosomal connections in *Escherichia coli*. Proc Natl Acad Sci U S A. 2012;109(28):11336–41. 10.1073/pnas.1208595109 22733746 PMC3396475

[156] Weickert MJ, Adhya S. The galactose regulon of *Escherichia coli*. Mol Microbiol. 1993;10(2):245–51. Epub 1993/10/01. 10.1111/j.1365-2958.1993.tb01950.x . 7934815

[157] Agerschou ED, Christiansen G, Schafer NP, Madsen DJ, Brodersen DE, Semsey S, et al The transcriptional regulator GalR self-assembles to form highly regular tubular structures. Sci Rep. 2016;6:27672 Epub 2016/06/10. 10.1038/srep27672 27279285 PMC4899725

[158] Bates D, Epstein J, Boye E, Fahrner K, Berg H, Kleckner N. The *Escherichia coli* baby cell column: a novel cell synchronization method provides new insight into the bacterial cell cycle. Mol Microbiol. 2005;57(2):380–91. 10.1111/j.1365-2958.2005.04693.x 15978072 PMC2973562

[159] Kavenoff R, Ryder OA. Electron microscopy of membrane-associated folded chromosomes of *Escherichia coli*. Chromosoma. 1976;55(1):13–25. 10.1007/bf00288323 . 767075

[160] Worcel A, Burgi E. Properties of a membrane-attached form of the folded chromosome of *Escherichia coli*. J Mol Biol. 1974;82(1):91–105. Epub 1974/01/05. 10.1016/0022-2836(74)90576-2 . 4594427

[161] Roggiani M, Goulian M. Chromosome-Membrane Interactions in Bacteria. Annu Rev Genet. 2015;49:115–29. 10.1146/annurev-genet-112414-054958 . 26436460

[162] Libby EA, Roggiani M, Goulian M. Membrane protein expression triggers chromosomal locus repositioning in bacteria. Proc Natl Acad Sci U S A. 2012;109(19):7445–50. Epub 2012/04/25. 10.1073/pnas.1109479109 22529375 PMC3358875

[163] Brameyer S, Rosch TC, El Andari J, Hoyer E, Schwarz J, Graumann PL, et al DNA-binding directs the localization of a membrane-integrated receptor of the ToxR family. Commun Biol. 2019;2:4 Epub 2019/02/12. 10.1038/s42003-018-0248-7 30740540 PMC6320335

[164] Espeli O, Borne R, Dupaigne P, Thiel A, Gigant E, Mercier R, et al A MatP-divisome interaction coordinates chromosome segregation with cell division in E. coli. EMBO J. 2012;31(14):3198–211. Epub 2012/05/15. 10.1038/emboj.2012.128 22580828 PMC3400007

[165] Bernhardt TG, de Boer PA. SlmA, a nucleoid-associated, FtsZ binding protein required for blocking septal ring assembly over Chromosomes in E. coli. Mol Cell. 2005;18(5):555–64. Epub 2005/05/27. 10.1016/j.molcel.2005.04.012 15916962 PMC4428309

[166] Almiron M, Link AJ, Furlong D, Kolter R. A novel DNA-binding protein with regulatory and protective roles in starved *Escherichia coli*. Genes Dev. 1992;6(12B):2646–54. 10.1101/gad.6.12b.2646 . 1340475

[167] Frenkiel-Krispin D, Ben-Avraham I, Englander J, Shimoni E, Wolf SG, Minsky A. Nucleoid restructuring in stationary-state bacteria. Mol Microbiol. 2004;51(2):395–405. 10.1046/j.1365-2958.2003.03855.x . 14756781

[168] Ball CA, Osuna R, Ferguson KC, Johnson RC. Dramatic Changes in Fis Levels Upon Nutrient Upshift in Escherichia-Coli. Journal of Bacteriology. 1992;174(24):8043–56. PubMed PMID: WOS:A1992KC71900023. 10.1128/jb.174.24.8043-8056.1992 1459953 PMC207543

[169] Claret L, Rouviere-Yaniv J. Variation in HU composition during growth of *Escherichia coli*: the heterodimer is required for long term survival. J Mol Biol. 1997;273(1):93–104. Epub 1997/11/21. 10.1006/jmbi.1997.1310 . 9367749

[170] Lal A, Dhar A, Trostel A, Kouzine F, Seshasayee AS, Adhya S. Genome scale patterns of supercoiling in a bacterial chromosome. Nat Commun. 2016;7:11055 10.1038/ncomms11055 27025941 PMC4820846

[171] Kar S, Edgar R, Adhya S. Nucleoid remodeling by an altered HU protein: reorganization of the transcription program. Proc Natl Acad Sci U S A. 2005;102(45):16397–402. Epub 2005/11/01. 10.1073/pnas.0508032102 16258062 PMC1283455

[172] Lim CJ, Lee SY, Kenney LJ, Yan J. Nucleoprotein filament formation is the structural basis for bacterial protein H-NS gene silencing. Sci Rep. 2012;2:509 10.1038/srep00509 22798986 PMC3396134

[173] Aki T, Choy HE, Adhya S. Histone-like protein HU as a specific transcriptional regulator: co-factor role in repression of gal transcription by GAL repressor. Genes Cells. 1996;1(2):179–88. 10.1046/j.1365-2443.1996.d01-236.x . 9140062

[174] Pagel JM, Winkelman JW, Adams CW, Hatfield GW. DNA topology-mediated regulation of transcription initiation from the tandem promoters of the ilvGMEDA operon of *Escherichia coli*. J Mol Biol. 1992;224(4):919–35. Epub 1992/04/20. 10.1016/0022-2836(92)90460-2 . 1569580

[175] Parekh BS, Hatfield GW. Transcriptional activation by protein-induced DNA bending: evidence for a DNA structural transmission model. Proc Natl Acad Sci U S A. 1996;93(3):1173–7. Epub 1996/02/06. 10.1073/pnas.93.3.1173 8577735 PMC40051

[176] Dorman CJ, Dorman MJ. DNA supercoiling is a fundamental regulatory principle in the control of bacterial gene expression. Biophysical reviews. 2016;8(3):209–20. Epub 2017/05/17. 10.1007/s12551-016-0205-y 28510224 PMC5425793

[177] Chong S, Chen C, Ge H, Xie XS. Mechanism of transcriptional bursting in bacteria. Cell. 2014;158(2):314–26. Epub 2014/07/19. 10.1016/j.cell.2014.05.038 25036631 PMC4105854

[178] Peter BJ, Arsuaga J, Breier AM, Khodursky AB, Brown PO, Cozzarelli NR. Genomic transcriptional response to loss of chromosomal supercoiling in *Escherichia coli*. Genome Biol. 2004;5(11):R87 Epub 2004/11/13. 10.1186/gb-2004-5-11-r87 15535863 PMC545778

[179] Berger M, Farcas A, Geertz M, Zhelyazkova P, Brix K, Travers A, et al Coordination of genomic structure and transcription by the main bacterial nucleoid-associated protein HU. EMBO reports. 2010;11(1):59–64. Epub 2009/12/17. 10.1038/embor.2009.232 20010798 PMC2816637

[180] Berger M, Gerganova V, Berger P, Rapiteanu R, Lisicovas V, Dobrindt U. Genes on a Wire: The Nucleoid-Associated Protein HU Insulates Transcription Units in Escherichia coli. Sci Rep. 2016;6:31512 10.1038/srep31512 27545593 PMC4992867

[181] Koli P, Sudan S, Fitzgerald D, Adhya S, Kar S. Conversion of commensal *Escherichia coli* K-12 to an invasive form via expression of a mutant histone-like protein. MBio. 2011;2(5). Epub 2011/09/08. 10.1128/mBio.00182-11 21896677 PMC3172693

[182] Kar S, Choi EJ, Guo F, Dimitriadis EK, Kotova SL, Adhya S. Right-handed DNA supercoiling by an octameric form of histone-like protein HU: modulation of cellular transcription. J Biol Chem. 2006;281(52):40144–53. Epub 2006/10/26. 10.1074/jbc.M605576200 . 17062578

[183] Vanhecke D, Graber W, Studer D. Close-to-native ultrastructural preservation by high pressure freezing. Method Cell Biol. 2008;88:151–64. 10.1016/S0091-679x(08)00409-3 PubMed PMID: WOS:000257693100009. 18617033

[184] Ou HD, Phan S, Deerinck TJ, Thor A, Ellisman MH, O'Shea CC. ChromEMT: Visualizing 3D chromatin structure and compaction in interphase and mitotic cells. Science. 2017;357(6349). 10.1126/science.aag0025 . 28751582 PMC5646685

[185] Narayan K, Subramaniam S. Focused ion beams in biology. Nat Methods. 2015;12(11):1021–31. Epub 2015/10/30. 10.1038/nmeth.3623 . 26513553 PMC6993138

