# Correction: Architecture of the *Escherichia coli* nucleoid

**DOI:** 10.1371/journal.pgen.1009148

**Published:** 2020-10-21

**Authors:** Subhash C. Verma, Zhong Qian, Sankar L. Adhya

There are two typographical errors in Fig 4. The authors have provided a corrected version here.

**Fig 4:**
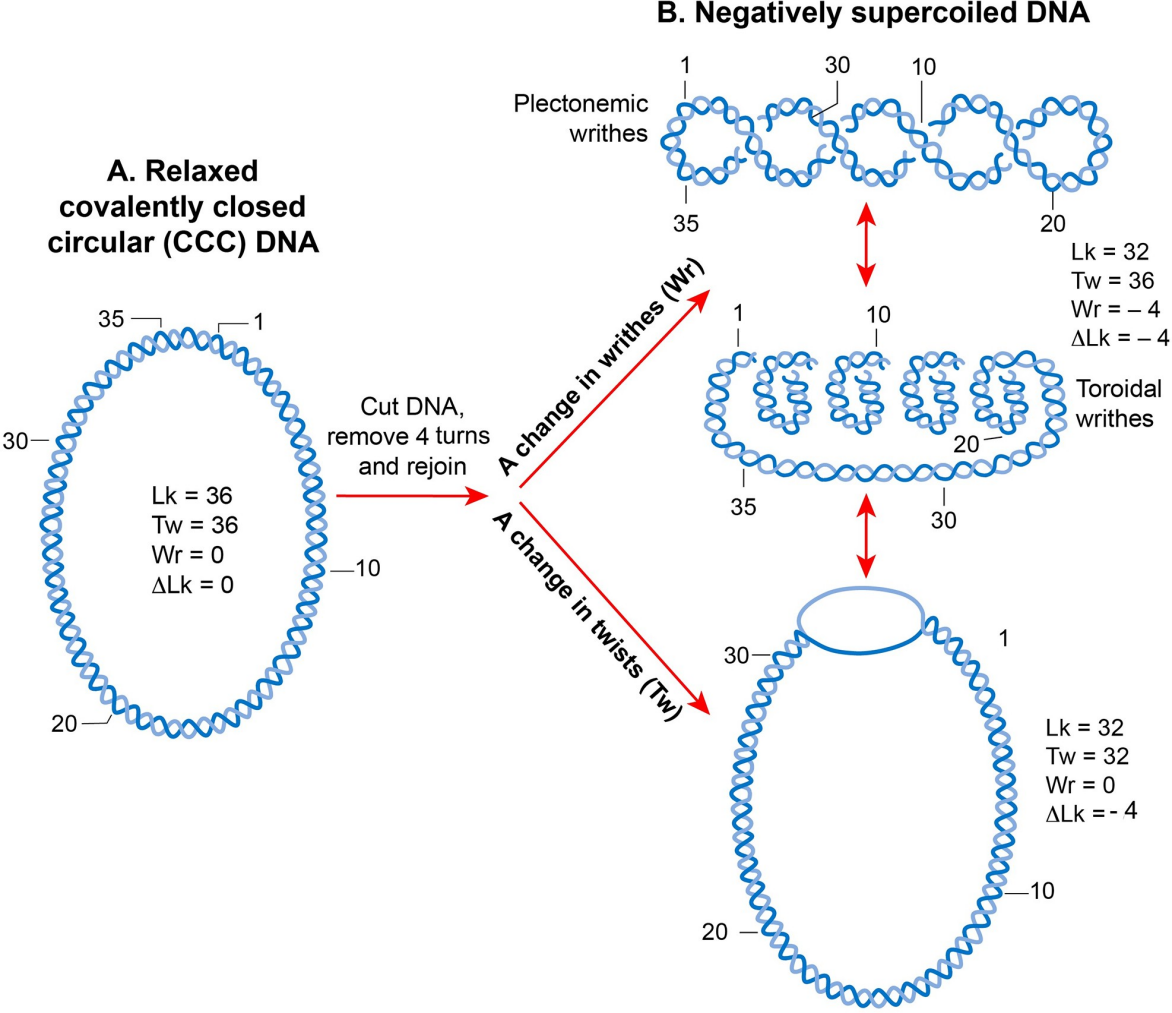
DNA supercoiling. **A**. A linear double-stranded DNA becomes a topologically constrained molecule if the two ends are covalently joined, forming a circle. Rules of DNA topology are explained using such a molecule (ccc-DNA) in which a numerical parameter called the linking number (Lk) defines the topology. Lk is a mathematical sum of two geometric parameters, twist (Tw) and writhe (Wr). A twist is the crossing of two strands, and writhe is coiling of the DNA double helix on its axis that requires bending. Lk is always an integer and remains invariant no matter how much the two strands are deformed. It can only be changed by introducing a break in one or both DNA strands by DNA metabolic enzymes called topoisomerases. **B**. A torsional strain created by a change in Lk of a relaxed, topologically constrained DNA manifests in the form of DNA supercoiling. A decrease in Lk (Lk<Lk_0_) induces negative supercoiling whereas an increase in Lk (Lk>Lk_0_) induces positive supercoiling. Only negative supercoiling is depicted here. For example, if a cut is introduced into a ccc-DNA and four turns are removed before rejoining the two strands, the DNA becomes negatively supercoiled with a decrease in the number of twists or writhe or both. Writhe can adopt two types of geometric structures called plectoneme and toroid. Plectonemes are characterized by the interwinding of the DNA double helix and an apical loop, whereas spiraling of DNA double helix around an axis forms toroids.

## References

[1] Verma SC, Qian Z, Adhya SL (2019) Architecture of the Escherichia coli nucleoid. PLoS Genet 15(12): e1008456 10.1371/journal.pgen.1008456 31830036 PMC6907758

